# Inactivation of tumor suppressor gene Clusterin leads to hyperactivation of TAK1-NF-κB signaling axis in lung cancer cells and denotes a therapeutic opportunity

**DOI:** 10.7150/thno.44829

**Published:** 2020-09-16

**Authors:** Zhipeng Chen, Zhenzhen Fan, Xiaowei Dou, Qian Zhou, Guandi Zeng, Lu Liu, Wensheng Chen, Ruirui Lan, Wanting Liu, Guoqing Ru, Lei Yu, Qing-Yu He, Liang Chen

**Affiliations:** 1 Key Laboratory of Functional Protein Research of Guangdong Higher Education Institutes and MOE Key Laboratory of Tumor Molecular Biology, Institute of Life and Health Engineering, College of Life Science and Technology, Jinan University, Guangzhou 510632, China.; 2 Clinical Research Center, the Affiliated Hospital of Guizhou Medical University, Guiyang 550004, China.; 3 Department of Pathology, Zhejiang, Provincial People's Hospital, People's Hospital of Hangzhou, Medical College, Hangzhou 310014, Zhejiang Province, China.; 4 Beijing Tongren Hospital, Capital Medical University, 100730 Beijing, China.

**Keywords:** precision medicine, tumor suppressor gene, non-small cell lung cancer, mouse model.

## Abstract

**Purpose:** Clinical success of precision medicine is severely limited by de novo or acquired drug resistance. It remains a clinically unmet need to treat these patients. Tumor suppressor genes (TSGs) play a critical role in tumorigenesis and impact the therapeutic effect of various treatments. **Experimental Design:** Using clinical data, *in vitro* cell line data and *in vivo* mouse model data, we revealed the tumor suppressive role of Clusterin in lung cancer. We also delineated the signaling cascade elicited by loss of function of CLU in NSCLC cells and tested precision medicine for *CLU* deficient lung cancers. **Results:** *CLU* is a potent and clinically relevant TSG in lung cancer. Mechanistically, CLU inhibits TGFBR1 to recruit TRAF6/TAB2/TAK1 complex and thus inhibits activation of TAK1- NF-κB signaling axis. Lung cancer cells with loss of function of CLU show exquisite sensitivity to TAK1 inhibitors. Importantly, we show that a significant portion of Kras mutation positive NSCLC patients are concurrently deficient of CLU and that TAK1 kinase inhibitor synergizes with existing drugs to treat this portion of lung cancers patients. **Conclusions:** Combinational treatment with TAK1 inhibitor and MEK1/2 inhibitor effectively shrank Kras mutation positive and CLU deficient NSCLC tumors. Moreover, we put forward a concept that loss of function of a TSG rewires signaling network and thereby creates an Achilles' heel in tumor cells which could be exploited in precision medicine.

## Introduction

The clinical success of cancer therapies is severely limited by de novo or acquired drug resistance. Accumulating lines of evidence showed that status of tumor suppressor genes significantly impacted the efficacy of precision medicine, including chemotherapy 1, targeting therapy 2 and immunotherapy 3. It remains an urgent and clinically unmet need to treat those cancer patients who are resistant to otherwise effective therapies in the presence of intact TSGs.

Lung cancer is the leading cause of cancer related deaths globally, with non-small cell lung cancer (NSCLC) the most frequently diagnosed pathological type of lung cancer. Chemotherapy, targeting therapy and immunotherapy are mainstream treatment options for lung cancer patients in clinic. Unfortunately, despite of the advances of multimodality of therapies, the prognosis for lung cancer patients remains disappointingly dismal with overall 5-year survival rate of only around 17% 4. The difficulties in developing effective therapies for lung cancer patients stem from our limited understanding of tumorigenesis of lung cancer.

Gain-of-function mutations in driver oncogenes and loss-of-functions in tumor suppressor genes (TSGs) are thought to coordinately drive the transformation of lung epithelial cells into tumorigenic cells 5, 6. Currently, driver oncogenes are relatively well-characterized, with multiple targeting drugs available for lung cancer patients in clinic 7, 8. However, TSGs remain to be systemically determined for lung cancer.

Clusterin was first identified as a highly conserved glycoprotein 9, encoded by the *CLU* gene locus on chromosome 8^10^. Functional study revealed *CLU* as a Golgi chaperone that facilitates the folding of secreted proteins in a manner similar to small heat shock proteins 10-12. It has been reported to be involved in numerous physiological processes including apoptotic cell death, cell cycle regulation, DNA repair, cell adhesion, tissue remodeling, lipid transportation, membrane recycling, and immune system regulation 13-15.

Emerging evidence supported *CLU* as a potent oncogene 16, consistent with reports showing its existence in exosomes and helping cancer cells to survive in distant locations 17. Overexpression of *CLU* has been reported in bladder cancer 18. Furthermore, ectopic expression of *CLU* in primary hepatocellular carcinoma cells increased migration by twofold *in vitro* and formation of metastatic tumor nodules in liver by eightfold *in vivo* 19, 20. Likewise, overexpression of *CLU* enhances the metastatic ability of human renal cell carcinoma 21 and prostate cancer 22. On the other hand, tumor suppressor function has also been reported for *CLU* in neuroblastomas 23, prostate cancer 24, and broadly epithelial cancers 25. Both tumor promoting or suppressing function have been reported for *CLU* in lung cancer 26-28. It, therefore, remains to be clarified *CLU*'s role in lung cancer.

Here we report that *CLU* is a potent and clinically relevant TSG in lung cancer. *CLU* inhibits lung cancer cell growth *in vitro* and tumorigenesis *in vivo*. Mechanistically, Clusterin inhibits TGFBR1 to recruit TRAF6/TAB2/TAK1 complex and thus inhibits activation of TAK1-NF-κB axis. Analysis of clinical expression data reveals that *CLU* is reversely correlated with expression of NF-κB target genes. In clinic, a significant portion of Kras mutation positive lung cancer patients concurrently harbored low level of* CLU* expression. We also show that TAK1 kinase inhibitor synergizes with existing drugs to treat this portion of Kras mutation positive lung cancers. Using lung cancer as a model, we show here that TSG dysfunction creates a targeting opportunity with potential for clinical application. Hereby, we put forward a concept that loss of function of a TSG significantly rewires signaling network and thereby creates an Achilles' heel in cancer cell, which could be exploited in precision medicine.

## Results

### *CLU* is an essential tumor suppressor in lung tumorigenesis

**Figure 1:**
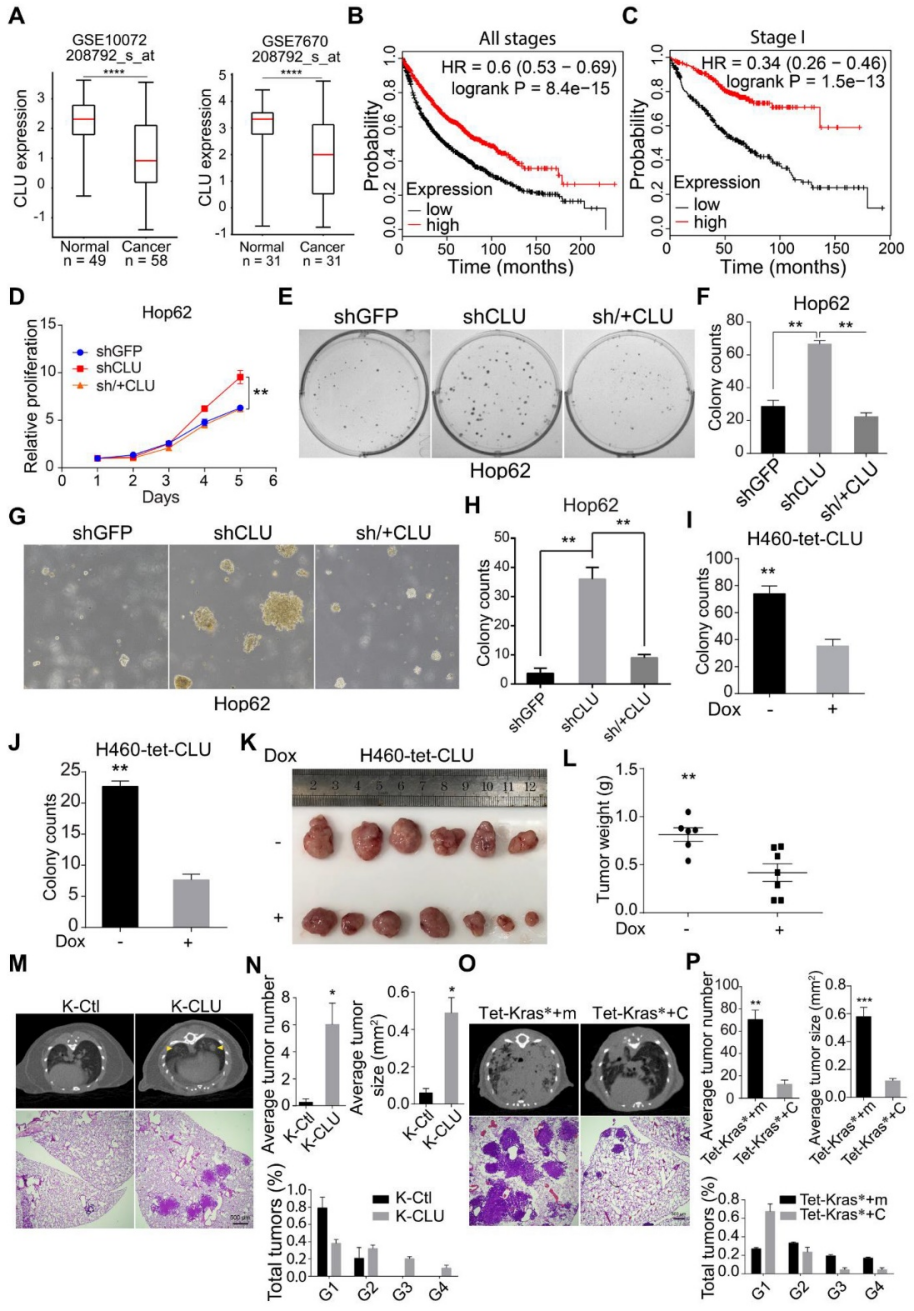
***CLU* is an essential tumor suppressor gene in lung cancer. (A)** Two published microarray data sets were analyzed to compare CLU expression in normal and tumoral tissues. GEO number and probe set were labeled on the graph. ****P < 0.00001.** (B)** and** (C)** Kaplan-Meier survival curve analysis of *CLU*-high and low lung cancer patients of all pathological stages **B** or stage I **C**. **(D)** Impact of *CLU* expression on growth rate of Hop62 cell. 800 engineered Hop62 cells were seeded in 96 wells plate and cultured for 5 days. Cell viability was analyzed with CCK8 assay. Statistic with two-tailed t-test on day 5. **(E)** Impact of *CLU* expression on 2-D colony formation ability of Hop62 cell. 200 engineered Hop62 cells were seed in 6 well plate and cultured for 7 days. Colonies were fixed and stained with 0.5% crystal violet in methyl alcohol. **(F)** Quantification of colony numbers of **E**. Statistic with one-way ANOVA test. **(G)** Impact of *CLU* expression on ability of Hop62 cell to form colonies in soft agar culture. 200 cells/well were seeded in 6-well plates and cultured for 14 days before imaging. **(H)** Quantification of **G**. shGFP for control knockdown; shCLU for CLU knockdown; sh/+CLU for *CLU* re-expression in *CLU* knockdown cells. **(I)** and **(J)** 2-D Plate and soft agar colony formation assay of indicated groups in H460 cell. CLU expression was induced with 1 ug/mL Dox. For 2-D colony formation assay, 200 cells/well were seed in 6 well plate and cultured for 7 days. Colonies were fixed with methanol and stained with 0.5% crystal violet. For soft agar colony formation, 200 cells/well were seeded and culture for 14 days before imaging. Two-tailed t-test. **(K)** Impact of *CLU* expression on ability of H460 to form xenograft tumor in nude mouse. H460 cells (2 million) were subcutaneously implanted on nude mice followed by Dox-diet treatment. Tumors were harvested 25 days post implantation. Each group n > 6.** (L)** Weight of tumor in **K**, two-tailed t-test.** (M)** Impact of *CLU* expression on tumor formation in lsl-Kras^G12D/+^ transgenic mice. Lentivirus of pSECC-sgCLU and pSECC-TdTomato were administered through nasal instillation into lsl-KrasG12D mice to induced lung cancer, designated K-CLU and K-Ctl respectively. Lung tumor formation in K-CLU mice were compared to K-Ctl mice 13 weeks post-infection. Upper panel: Computed tomography (CT) images of lung of Kras^G12D/+^ mouse; Lower panel: hematoxylin and eosin (H&E) staining of lung sections of Kras^G12D/+^ mouse. K-Ctl for Kras^G12D/+^/sgControl; K-CLU is Kras^G12D/+^/sgCLU, each group n > 6.** (N)** Quantification of average total tumor number, tumor size and the percentage of G1-G4 tumors for **M**.** (O)** Impact of CLU expression on tumor formation in CC10rtTA /Tet-Kras^G12D^ transgenic mice. Upper panel: Computed tomography images of lung of CC10rtTA /Tet-Kras^G12D^ transgenic mice; Lower panel: H&E staining of lung sections of Tet-Kras^G12D^ transgenic mice. CC10rtTA/ Tet-Kras^G12D^ mice infected with lentivirus harboring Tet-mCherry (Tet-Kras*+m, serving as negative control) or lentivirus harboring Tet-CLU (Tet-Kras*+C) were fed with Dox diet for 2 months. Each group n > 6. **(P)** Quantification of average total tumor number, tumor size and percentage of G1-G4 tumors of **O**, statistics with two-tailed t-test. All the transgenic mice here were C57BL6 background, about 6-8 weeks old without sex limited. *P < 0.05, **P < 0.005, ***P < 0.0001; Data plotted are mean ± s.e.m.; n = 3.

In our previous systemic *in vivo* screening of lung cancer TSGs, we noticed that somatic knockout of *CLU* in pulmonary epithelia promoted lung cancer development, suggesting *CLU* to be a TSG in lung cancer 29. To find out clinical evidence for *CLU* as a TSG in lung cancer, we compared CLU expression level in lung adenocarcinoma against para-tumoral tissues using GEO data sets (GSE10072 and GSE7670) downloaded from NCBI GEO database and found significantly lower levels of *CLU* in NSCLS tissues (Figure 1A). We also analyzed *CLU* mRNA level in lung cancer patients using XENA online tool (http://xena.ucsc.edu/compare-tissue/), which integrated all published comparable data set for expression level analysis, and found significantly lower levels in lung cancers than in normal or para-tumoral lung tissues (Figure S1A and Table S1). Moreover, a higher level of *CLU* was significantly associated with patients' longer overall survival (Figure 1B). We also noticed similar significant correlation in stage I patients (Figure 1C), indicating that *CLU* was a clinically relevant TSG in lung cancer and that *CLU* played an essential role in early stage of lung cancer development.

Two functionally different splicing variants of *CLU* transcripts have been reported, namely full-length *CLU* (sCLU) and truncated *CLU* (nCLU). To find out which variant is downregulated in lung cancer samples, we analyzed their expression in lung cancer samples, para-tumoral tissues and NSCLC cell lines through semi-quantitative RT-PCR with a single pair of primers capable of amplifying both variants and separated the PCR products through high-resolution agarose-gel electrophoresis. Based on the size of the resolved PCR product, we found that only the full-length *CLU* was expressed in all these samples and that its expression was relatively lower in cancer cells and lung cancer samples in comparison to para-tumoral tissues (Figure S1B). Sanger sequencing confirmed authenticity of sCLU (Figure S1C). sCLU is translated into a protein of around 64 kDa, which is subjected to proteolysis between Arg^205^ and Ser^206^ to generates α- and β-chains; α- and β-chains are then linked by five interchain disulfide bonds, which is secreted into extracellular space 9. Consistently, our western analysis revealed both 64-kDa full-length and 40-kDa CLU variants in total cell lysates and mainly the 40-kDa cleaved fragment in supernatant (Figure S1D). Based on these data, we focused our further experimental efforts on sCLU (the full-length variant, and hereafter designated *CLU* unless specified) for studying tumor suppressive function in lung cancer.

In order to validate its TSG function, we sought to ectopically express *CLU* in lung cancer cell lines with lower baseline expression and knockdown in those with relatively higher expression. For this purpose, we checked *CLU* expression level in lung cancer cell lines commonly used in cancer research community through qRT-PCR analysis, including two pairs of lung cancer/para-tumoral tissues as references for *CLU* expression in tumoral and normal lung tissues. We found comparable *CLU* mRNA level between tumor samples and most of the lung cancer cell lines, which was consistently lower than that of para-tumoral tissues (Figure S1E). Similar pattern was seen in CLU protein level (Figure S1F). We pick A549 and HOP62 as CLU-high and H460 and EKVX CLU-low cells. We then knockdown *CLU* in Hop62 cells (designated Hop62-shCLU) and found that *CLU* knockdown significantly promoted growth rate as compared to control knockdown cells (Hop62-shGFP). Moreover, re-expression of shRNA resistant *CLU* (designated Hop62-sh/+CLU) slowed down the growth rate to the degree comparable to that of Hop62-shGFP (Figure S1G & Figure 1D). We also found that *CLU* knockdown enhanced the capacity of Hop62 cells to form colonies in 2-D plate and that ectopic expression of shRNA-resistant *CLU* downregulated this colony-forming ability (Figure 1E-F). We found similar pattern of colony-forming ability in soft agar culture (Figure 1G-H). These effects were repeated on A549 cells (Figure S1H-K), strongly arguing TSG function for *CLU* in lung cancer. We also generated H460 and EKVX cells for doxycycline (Dox) inducible expression of *CLU* (designated H460-tet-CLU and EKVX-tet-CLU, Figure S1L). We found that Dox treatment inhibited the growth rate (Figure S1M), ability to form colonies in 2-D plates (Figure 1I & Figure S1N) and soft-agar culture conditions for H460 cells (Figure 1J & Figure S1O). Similar effect was also found using EKVX-tet-CLU cell line (Figure S1P-R). These *in vitro* data strongly argued that *CLU* was a potent TSG in lung cancer.

To evaluate the tumor suppressive role of *CLU in vivo*, we used xenograft tumor model. We found that expression of *CLU* significantly suppressed growth of xenograft tumor derived from H460-tet-CLU (Figure 1K-L).

Mouse models of autochthonous lung cancer recapitulate the course of tumorigenesis and tumor development more faithfully than xenograft tumor models. We then went on to study the tumor suppressive function of *CLU* using mouse models of autochthonous lung cancer. Jacks and colleagues have earlier established an efficient method for simultaneous knockout of a target gene and activation of mutant Kras in the lung epithelia of lsl-Kras^G12D^ transgenic mice using recombinant lentivirus co-expressing Cre and CRISPR/CAS9^30^. Following this protocol, we intranasally delivered lentiviruses targeting either TdTomato (serving as negative control) or *CLU* (*CLU* knockout efficiency in Figure S1S) into lsl-Kras^G12D^ mice. Lsl-Kras^G12D^ mice infected with sgTdTomato virus (designated K-Ctl for control Kras^G12D^ mice) look rather healthy 13 weeks after treatment. Computed tomography (CT) imaging revealed little tumor burden at this stage (Figure 1M). Consistently, pathological analysis revealed occasional macroscopic lung tumor nodules of lung adenoma (Figure 1M). In stark contrast, mice began panting and exhibited hunched posture post 13 weeks of nasal inhalation of lenti-sgCLU (referred to as K-CLU mice for **K**ras^G12D^/***CLU***-/-), indicative of severe lung disease. CT imaging confirmed relatively heavier tumor burden in K-CLU mice (Figure 1M). Consistently, we detected macroscopic lung tumor nodules in 4 out of 6 mice (Figure 1M). Statistical analyses showed that *CLU* deletion significantly increased mutant Kras-driven lung tumor numbers, tumor size and amount of stage 4 tumors (Figure 1N).

We also studied the impact of *CLU* overexpression on tumorigenesis using Dox inducible Tet-Kras^G12D^/CC10rtTA mice (referred to hereafter as Tet-Kras* mice). Tet-Kras* mice develop lung cancers after feeding with Dox diet for around 3 months, but remain lung cancer free if fed with normal diet 31. We then infected lung epithelial compartment of Tet-Kras* mice with lentivirus harboring Tet-CLU cassette through intra-nasal instillation (designated Tet-Kras*+C mice) following our earlier protocol 32, such that virus-infected mice started to express Kras^G12D^ and *CLU* in lung epithelial cells when fed with Dox diet (Figure S1T). In parallel, we also generated a cohort of Tet-Kras* mice infected with lentivirus harboring Tet-mCherry (serving as Control, designated Tet-Kras*+m) (Figure S1T). Tet-Kras*+m mice began panting and exhibited hunched posture 3 months after Dox diet treatment, suggestive of severe lung disease. CT imaging revealed heavy tumor burdens in both lungs of these mice at this stage. Pathological analysis revealed poorly differentiated lung adenocarcinomas with features of diffused bronchial adenocarcinomas (Figure 1O). In stark contrast, Tet-Kras*+C mice looked largely normal at this stage and harbored significantly lower burden of lung cancers (Figure 1O), as well as tumor number and tumor size (Figure 1P). Moreover, significantly lower percentage of malignant tumors were found in Tet-Kras*+C mice model (Figure 1P).

Of note, *in vitro* and *in vivo* tumor suppressive functions of CLU were also confirmed on lung cancers driven by two other important oncoproteins, namely mutant EGFR driven and EML4-ALK driven lung cancers (Figure S2A-G).

Taken together, our data strongly argued that *CLU* was a clinically relevant and essential TSG in lung cancer.

### CLU knockdown enhances TAK1 signaling

**Figure 2:**
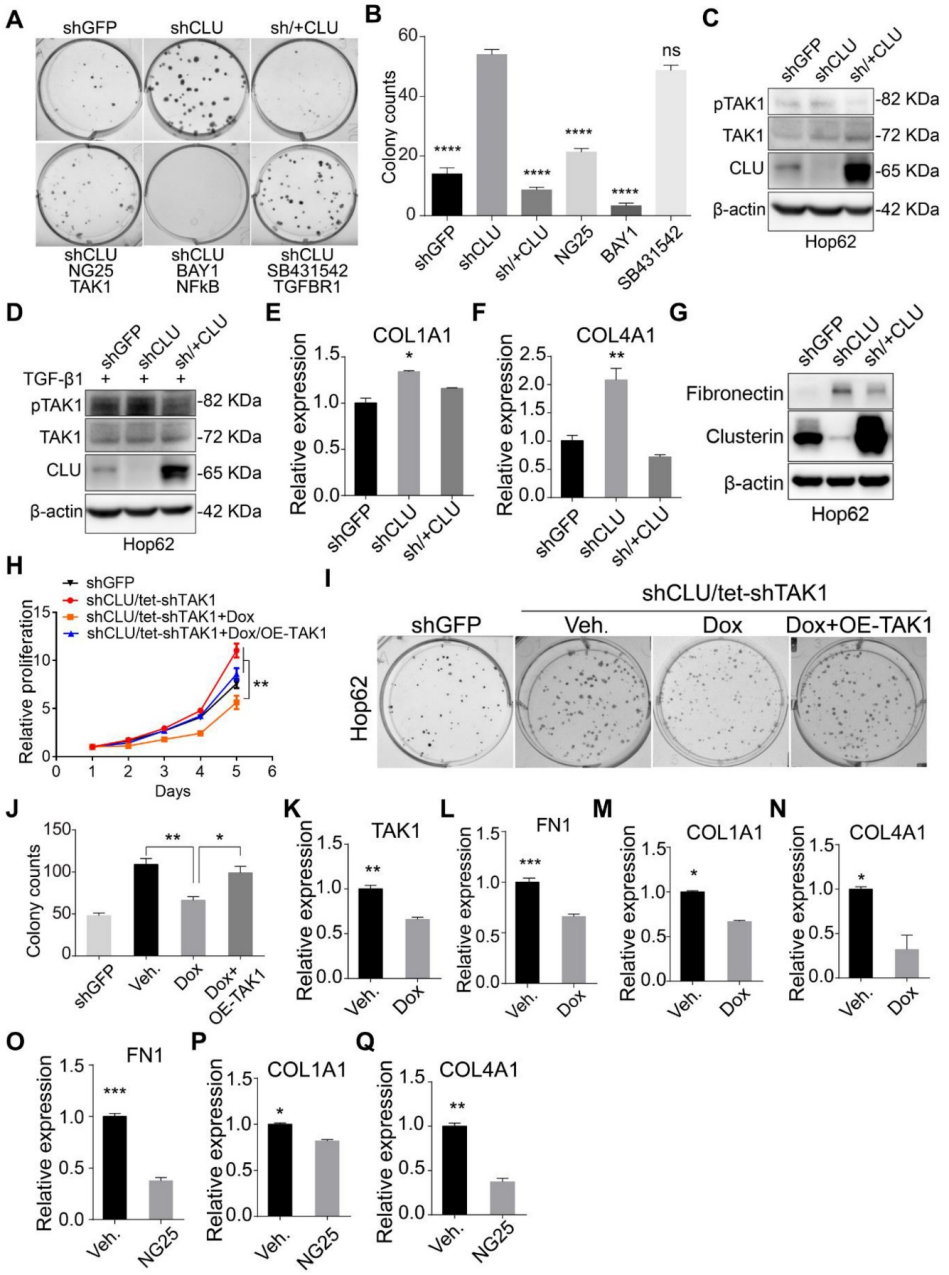
***CLU* knockdown enhances TAK1 signaling. (A)** TAK1 or NF-κB inhibitor specifically reduced colony forming ability of *CLU* knockdown lung cancer cell. 200 Hop62-shCLU cells were seeded and treated with indicated inhibitors for 7 days; NG25 (2.5 μM), BAY001 (1 μM), SB431542 (2.5 μM). Cell colonies were fixed and stained with 0.5% crystal violet in methyl alcohol. shGFP for control knockdown; shCLU for CLU knockdown; sh/+CLU for CLU re-expression in CLU knockdown cells.** (B)** Quantification of colony numbers. Statistic with one-way ANOVA test. **(C)** and **(D)** Impact of *CLU* expression on TAK1 activity. Hop62 cells were treated** (C)** without or **(D)** with TGF-β1 (5 ng/mL) for 2 h. Phosphor-TAK1, total TAK1 and CLU protein level was detected with indicated antibodies. **(E)** and **(F)** RT-PCR analysis of impact of CLU knockdown or re-expression on COL1A1 and COL4A1 expression in Hop62 cells. Total RNA of Hop62 cells with indicated were extracted and COL1A1 and COL4A1 expression was detected with RT-PCR.** (G)** Impact of *CLU* expression on Fibronectin expression in lung cancer cells. Fibronectin and CLU levels were assayed through Western analysis. **(H)** Impact of TAK1 knockdown or re-expression on cell proliferation of CLU knockdown Hop62 (Hop62-shCLU/tet-shTAK1) cells. TAK1 knockdown in Hop62-shCLU cells was induced with 1 μg/mL Dox. Statistic shown on day 5 with two-tailed t-test. **(I)** Impact of TAK1 knockdown on 2-D colony formation of CLU knockdown Hop62 cells. Cell colonies were fixed and stained with 0.5% crystal violet in methyl alcohol. **(J)** Quantification of **I**, one-way ANOVA test.** (K-N)** RT-PCR analysis of impact of TAK1 knockdown on *FN1*, *COL1A1* and *COL4A1* expression in *CLU* knockdown Hop62 cells. Hop62-shCLU cells were treated with 1 μg/mL Dox for TAK1 inducible knockdown. Statistic with two-tailed t-test. **(O-Q)** RT-PCR analysis of the impact of TAK1 inhibition with NG25 on *FN1*, *COL1A1* and *COL4A1* expression. Hop62-shCLU cells were treated with NG25 (2.5 μM) for 24 h before detecting mRNA level. *P < 0.05, **P < 0.005, ***P < 0.0001; Data plotted are mean ± s.e.m.; n = 3.

Having confirmed *CLU*'s growth inhibitory effect on lung cancer cells, we went on to study the underlying mechanism. We tried to find out the signaling pathway responsible for enhanced growth rate caused by *CLU* knockdown. For this purpose, we checked the impact of inhibitors against various growth promoting pathways on the growth rate of Hop62-shCLU. Our pilot experiment showed that *CLU* re-expression almost completely neutralized the enhanced ability to form colonies on 2-D plate caused by *CLU* knockdown, suggesting that 2-D colony forming assay was suitable for evaluating the growth inhibiting ability of a particular signaling pathway (Figure 2A). We then tested inhibitors against signaling pathways frequently involved in cell growth, including TGFBR (SB431542), PI3K (LY294002), AKT (MK2206), NOTCH (RO4929097), JAK (Ruxolitinib), EGFR (Gefitinib), NF-κB (BAY001) and TAK1 (NG25) and found inhibitors against NF-κB and TAK1 were the most potent hits in our screening (Figure 2A-B & Figure S3A-B). As TAK1 is an upstream activator of NF-κB^33^, our result suggested that TAK1-NF-κB signaling axis mediated the growth promoting effect caused by *CLU* knockdown. In line with this, phosphorylation of TAK1 is higher in Hop62-shCLU, but lower when re-expressed *CLU* (Figure 2C). Interestingly, although TGF-β1 treated Hop62 cells exhibited higher baseline level of TAK1 phosphorylation, we also found the ability of Clusterin to inhibit TAK1 phosphorylation (Figure 2D), suggesting that Clusterin regulated TAK1 activity through a mechanism parallel to TGF-β1 stimulation. Fibronectin (encoded by *FN1* gene), COL1A1 and COL4A1 expression have been reported to be positively regulated by TAK1 activity 34, 35. Consistently, quantitative RT-PCR confirmed that COL1A1 and COL4A1 were negatively regulated by CLU (Figure 2E-F). We also found that *CLU* knockdown increased and its re-expression downregulated Fibronectin expression in Hop62 cell (Figure 2G). Similar results were found in A549 and EKVX lung cancer cells (Figure S3C-D). Critically, we found that TAK1 knockdown prevented the enhancement of proliferation of Hop62 cells caused by *CLU* knockdown (Figure S3E & Figure 2H). Similar pattern was observed in 2-D colony forming ability of Hop62 (Figure 2I-J). Moreover, TAK1 knockdown or NG25 treatment decreased *FN1, COL1A1* and *COL4A1* expression in Hop62-shCLU cells (Figure 2K-Q). Taken together, our data showed that Clusterin negatively regulated TAK1's activity to promote growth of NSCLC cells.

### Clusterin inhibits TGFBR1 to recruit TRAF6/TAB2/TAK1 complex

**Figure 3:**
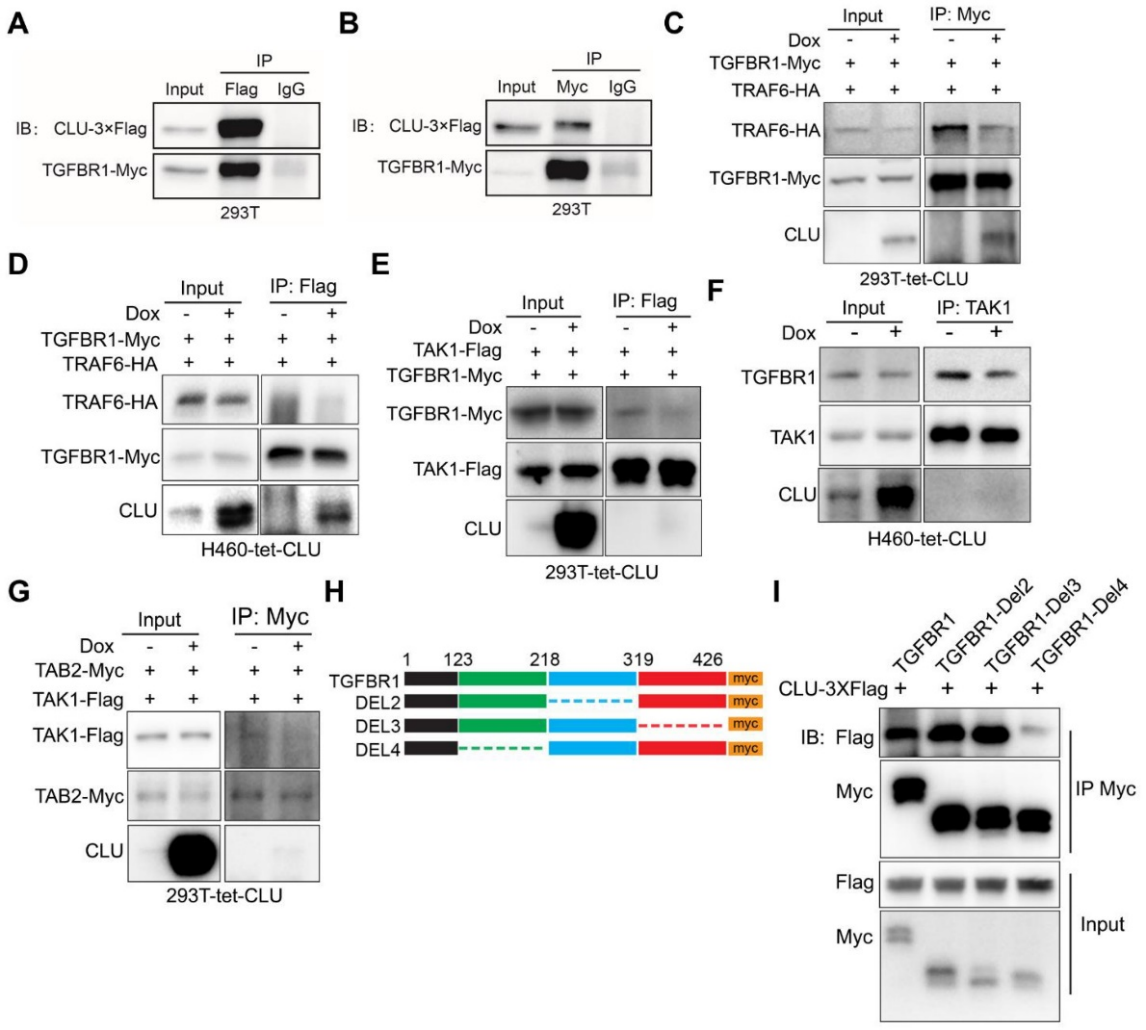
** CLU competes against TAK1 for binding TGFBR1. (A)** and **(B)** Reciprocal immunoprecipitation showing interaction between TGFBR1 and CLU. 293T cells were co-transfected with CLU-3×Flag and TGFBR1-myc expressing plasmid, followed by Co-IP with Flag **(A)** or myc **(B)** antibody. **(C)** 293T-tet-CLU cells were co-transferred with TRAF6-HA and TGFBR1-Myc expressing plasmids as indicated. Cell lysates were IPed with Myc antibody followed by western blotting with HA, Myc and CLU antibodies respectively. **(D)** H460-tet-CLU cells were co-transferred with TRAF6-HA and TGFBR1-Myc expressing plasmids as indicated. Cell lysates were IPed with Myc antibody followed by western blotting with HA, Myc and CLU antibodies respectively.** (E)** 293T-tet-CLU cell were co-transfected with TAK1-Flag and TGFBR1-Myc expressing plasmids. Cell lysates IPed with Flag antibody followed by western blotting with Myc, Flag and CLU antibodies respectively.** (F)** H460-tet-CLU cell lysate IPed with TAK1 antibody followed by western blotting with TGFBR1, TAK1 and CLU antibodies. **(G)** 293T-tet-CLU cells were co-transfected with TAK-Flag and TAB2-Myc expressing plasmids as indicated. Cell lysates were IPed with Myc antibody followed by western blotting with Flag, Myc and CLU antibodies respectively. **(H)** Schematic diagram of TGFBR1-myc truncation mutation. **(I)** Co-IP evaluating the interaction between CLU and TGFBR1 or its mutants. 293T cells were co-transfected with CLU-3×Flag and TGFBR1-myc or its muctant expressing plasmid for 24 h before performing Co-IP with Myc antibody and immunoblotting with Flag and Myc tag antibodies.

We then asked the molecular mechanism underlying the ability of Clusterin to negatively regulate TAK1 activity. Earlier, genome-wide screening has shown that Clusterin interacted with TGFBR1^36^. We were able to confirm interaction between Clusterin and TGFBR1 when overexpressed in 293T cells (Figure 3A & 3B).

Considering the facts that TGFBR1 directly recruits TRAF6^37^ and that TAB2 recruit TAK1 to TRAF6 through direct interaction with both protein 38, we hypothesized that CLU inhibited TGFBR1 to recruit TRAF6/TAB2/TAK1 complex in lung cancer cells, and thereby inhibit the signaling of downstream TAK1-NF-κB signaling pathway.

To test above hypothesis, we generated a 293T cell line for Dox inducible expression of Clusterin (designated 293T-tet-CLU, Figure S4A). Bimolecular fluorescence complementation (BiFC) assay enables efficient visualization of interactions between two proteins in cells by fusing both target proteins to C- and N- terminal half of luciferase respectively 39. We then conducted BiFC assay in 293T-tet-CLU cell line and found that CLU expression inhibited interaction between TGFBR1 and TRAF6 (Figure S4B). Co-immunoprecipitation (co-IP) further confirmed the ability of CLU to inhibit interaction between ectopically overexpressed TRAF6 and TGFBR1 in 293T-tet-CLU cell (Figure 3C). To test whether this was also true in lung cancer cells, we generated H460 for Dox inducible expression of Clusterin (designated H460-tet-CLU, Figure S1L) and found that CLU expression effectively inhibited recruitment of TRAF6 by TGFBR1 (Figure 3D).

If CLU blocked recruitment of TRAF6 by TGFBR1, TGFBR1/TRAF6/TAB2/TAK1 complex is expected to fall apart in cells expressing CLU. Consistent with our hypothesis, BiFC assay revealed that CLU inhibited interaction between TGFBR1 and TAB2 (Figure S4C); TGFBR1 and TAK1 (Figure S4D); TAK1 and TRAF6 (Figure S4E), and TAK1 and TAB2 (Figure S4F). More importantly, co-IP experiment confirmed that CLU inhibited the interaction between TGFBR1 and TAK1 in 293T-tet-CLU (Figure 3D) and H460-tet-CLU (Figure 3F) or TAK1 and TAB2 in 293T-tet-CLU (Figure 3G). Collectively, our data strongly argued that CLU expression inhibited TGFBR1 to recruit TRAF6/TAB2/TAK1 complex.

To pin down the domain of TGFBR1 important for interacting with Clusterin, we went on to generate a series of truncation mutants of TGFBR1. Intracellular part of TGFBR1 features 2 domains: GS domain of 30 amino acids (aa) and kinase domain of 291 aa. In considering this pattern of unbalanced distribution, we then constructed a series of mutation with approximately 100 aa deletion (Figure 3H). Our co-IP result showed that 123-218aa region of TGFBR1 is critical for binding to Clusterin, as mutant deleted of this region showed very weak interaction with Clusterin (Figure 3I).

### TAK1-NF-κB pathway mediates growth-promoting effects in *CLU*-deficient lung cancer cells

**Figure 4:**
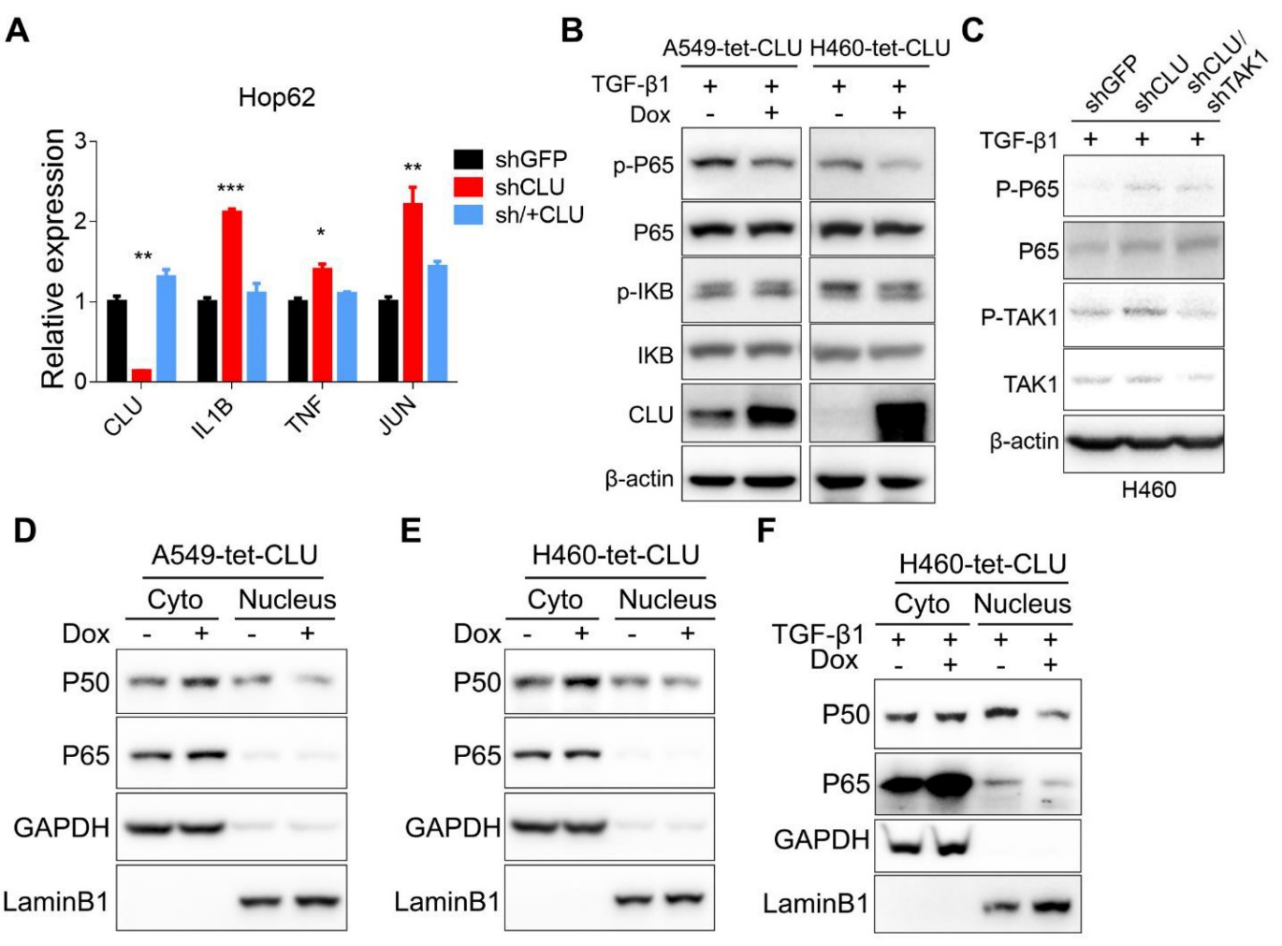
** TAK1-NF-κB pathway mediates growth-promoting effects in CLU-deficient lung cancer cells. (A)** RT-PCR evaluation of expression of NF-κB target genes in CLU knockdown or replenished Hop62 cells. Total RNA of engineered Hop62 cells were extracted. Expression of indicated genes was assayed with RT-PCR. **(B)** Influence of CLU expression on phosphorylation of NF-κB proteins in lung cancer cells. Lung cancer cell lines were treated with TGF-β1 with or without Dox. Total proteins were separated by SDS-PAGE following immunoblotting with antibodies against indicated NF-κB proteins. **(C)** Western blot evaluating the impact of TAK1 on P65 activation in CLU knockdown cells. Cells were treated with TGF-β1 (5 ng/mL) for 4 h. **(D)** and **(E)** Western blot evaluating the impact of CLU on cellular localization of NF-κB proteins. CLU expression in engineered lung cancer cells was induced with Dox. Cytoplasmic and nuclear fractions were separated from the lung cancer cells, followed by western blot with indicated antibodies. **(F)** Lung cancer cells were treated with TGF-β1 (5 ng/mL) for 2 hours before cytoplasmic and nuclear fraction were separated. Western blot was performed with indicated antibodies. Loading control: GAPDH for cytoplasmic; LaminB1 for nuclear. *P < 0.05, **P < 0.005, ***P < 0.0005, n = 3.

TAK1 signals through MKK3/P38 and NF-κB branches, both of which play important roles in cell growth 34. We detected no significant impact of p38 inhibitor on the growth of Hop62-shCLU (Figure S5A-B), suggesting that this branch might not play an important role in mediating growth-promoting effect in *CLU* knockdown NSCLC cells. On the other hand, our inhibitor screening experiment has shown that NF-κB inhibitor BAY001 or TAK1 inhibitor NG25 significantly inhibited colony formation of HOP62-shCLU (Figure 2A), suggesting that *CLU* inhibited lung cancer cells by negatively regulating TAK1-NF-κB signal axis. Consistently, we detected a significant increase in mRNA expression of NF-κB transcriptional targets in HOP62-shCLU cells, including IL-1β, TNF and Jun, which were inhibited by re-expression of *CLU* (Figure 4A). Likewise, overexpression of *CLU* inhibited the increased P65 phosphorylation caused by TGF-β1 treatment in H460. Of note, this effect was also seen in A549 (Figure 4B). Importantly, knockdown the expression of TAK1 prevented the activation of P65 in *CLU* knockdown H460 cells (Figure 4C).

Activation of the IκB kinase (IKK) complex induces the phosphorylation and subsequent degradation of IκBs, resulting in releasing P50 and P65 complex to translocate into the nucleus to function as transcription factors. Consistently, we observed an increase of cytoplasmic and concurrent decrease of nuclear P50 and P65 in A549-tet-CLU (Figure 4D) and H460-tet-CLU cells (Figure 4E) after Dox treatment. Of note, we observed similar impact of *CLU* expression on NF-κB signaling in H460 cultured in the presence of TGF-β1 (Figure 4F).

Taken together, our data showed that TAK1-NF-κB axis played a critical role mediating growth-promoting effect of *CLU*-deficient lung cancer cells.

### CLU-TAK1-NF-κB signaling axis is clinically relevant

**Figure 5:**
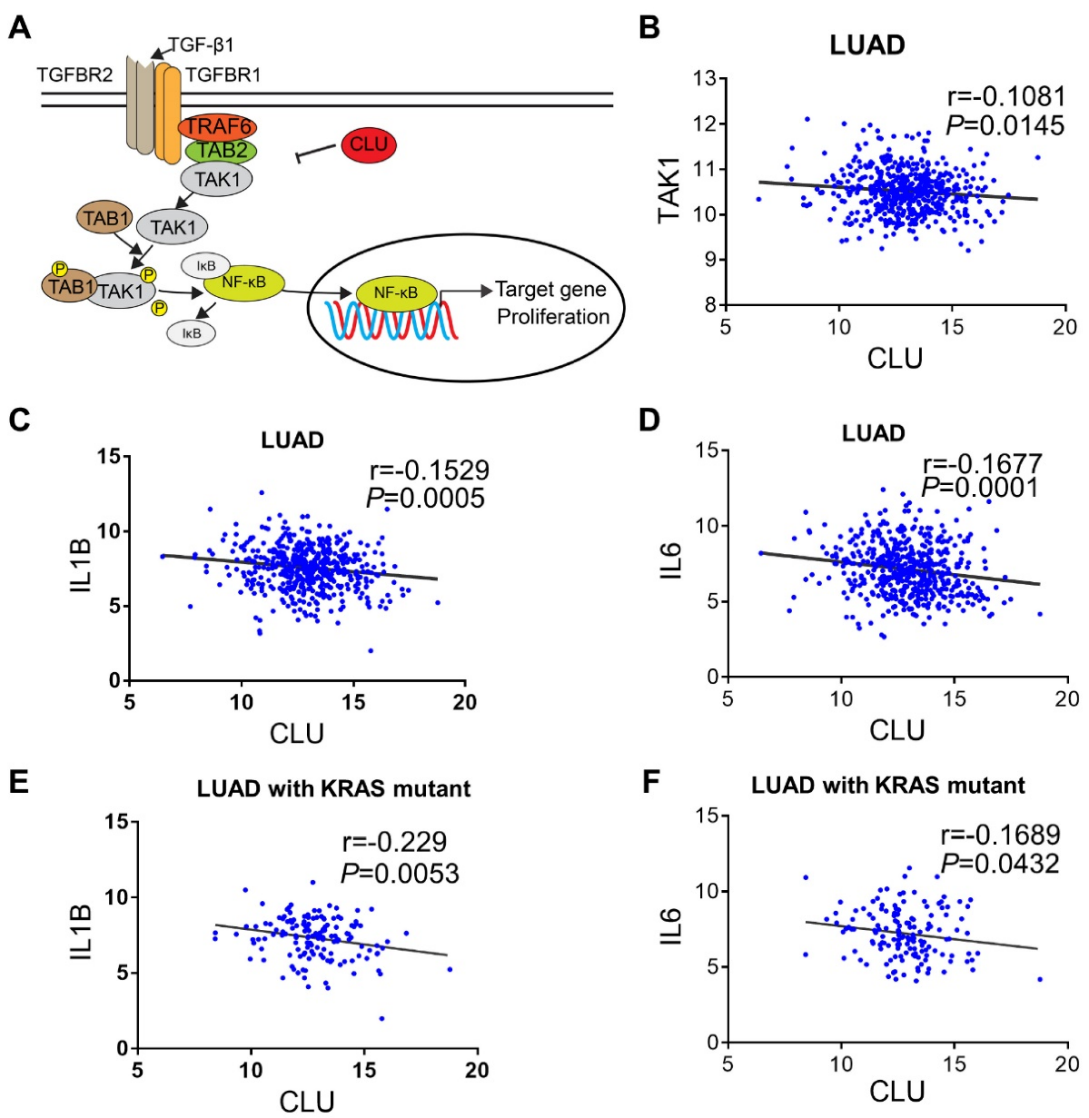
** CLU-TAK1-NF-κB signaling axis is clinically relevant. (A)** Schematic model of CLU-TAK1-NF-κB signaling axis in lung cancer. **(B)** Correlation analysis of expression between CLU and TAK1. Expression data of lung adenocarcinoma cancer patients from TCGA data base (analyzed through UCSC Xena). **(C)** and** (D)** Correlation analysis between CLU and NF-κB target gene IL1B and IL6.** (E)** and **(F)** Correlation analysis between CLU and NF-κB target gene IL1B and IL6 in lung adenocarcinoma cancer patients positive of *KRAS* mutation. Pearson r and P value are shown in images. Date are presented as log2 RSEM. *P* value and Person r were shown on the figure.

Based on our signaling model (Figure 5A), we predicted that CLU protein level reversely correlated with NF-κB signaling activity in lung cancer cells. Interestingly, we found significant reverse correlation between CLU and TAK1 (Figure 5B). We also detected reverse correlation between CLU and IL1β or IL6, two important NF-κB target genes (Figure 5C-D).

NSCLC are known to be driven by oncogenic alterations 40. Interestingly, we found that approximately 80% of KRAS mutation positive NSCLC patient concurrently had diminished CLU expression (Table S2). Likewise, expression level of CLU was significantly reverse-correlated with that of IL1β or IL6 in these *KRAS* mutation positive patients (Figure 5E-F).

### TAK1 inhibitor synergizes with existing therapeutics to treat CLU deficient lung cancer

Our above data have shown that a portion of lung cancer patients are concurrently Kras mutation positive and CLU-deficient (designated K+/C- patients). We then went on to test whether TAK1 inhibitor may synergize with existing drugs for treating this portion of lung cancers. We chose H358 cells (Kras^G12C^) to model K+/C- lung cancer patients (Figure S1F).

**Figure 6:**
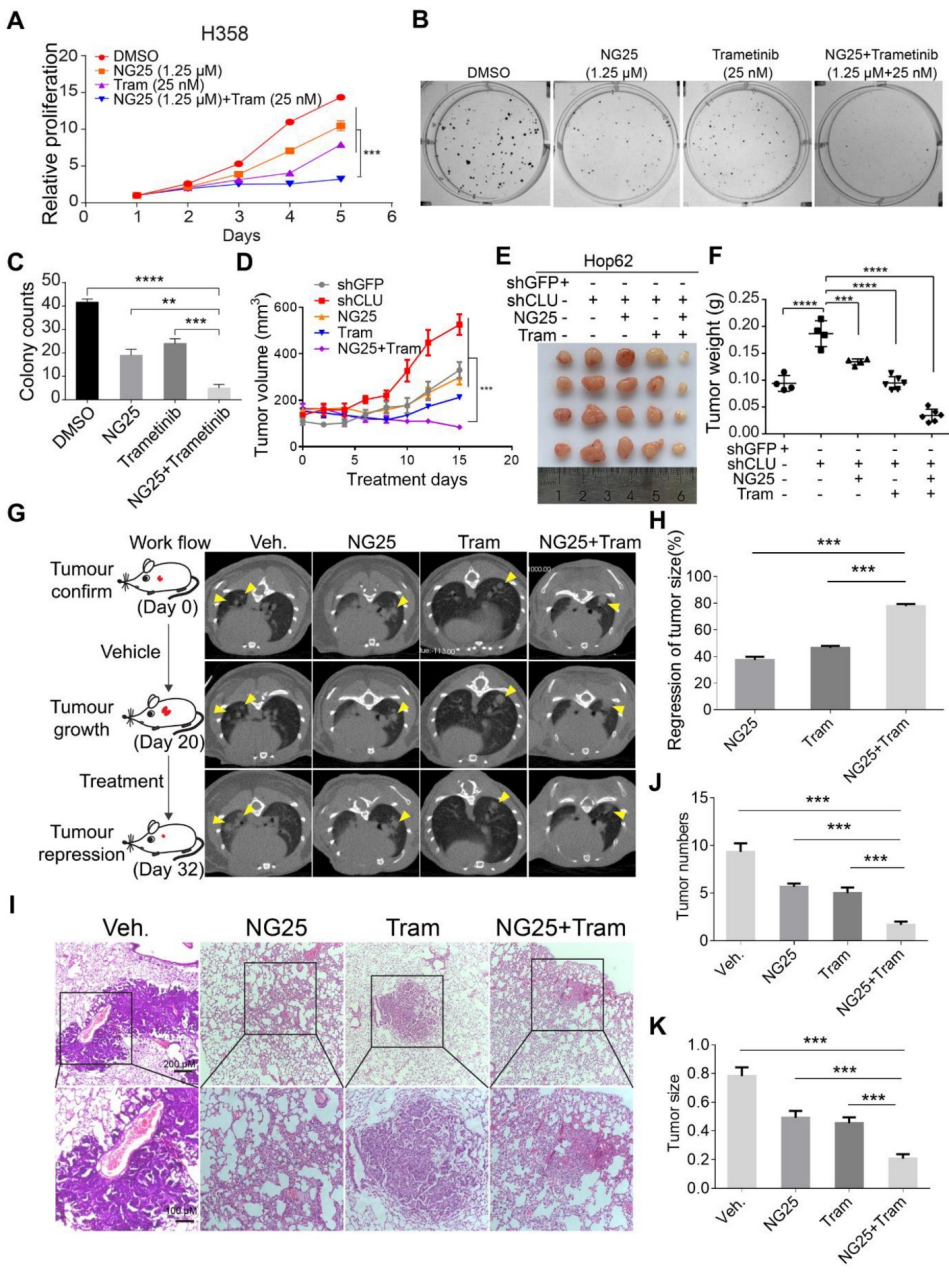
** TAK1 inhibitor synergizes with existing therapeutics to treat CLU deficient lung cancer. (A)** NG25 synergizes with Trametinib to inhibit growth of H358 cells. 200 cells/well were seeded in 96 wells plate and cultured for 5 days with indicated treatment. Viability of cells analyzed with CCK8 assay. Statistics done on day 5 with two-tailed t-test. **(B)** NG25 synergizes with Trametinib to inhibit 2-D colony formation of H358 cells. Cells were culture for 7 days with indicated treatment. **(C)** Quantification of **B**, one-way ANOVA test. **(D)** NG25 synergizes with Trametinib to inhibit growth of Hop62-shCLU derived xenograft tumor. Hop62-shCLU cells (3 million) were s.c. implanted in the flanks of nude mice. 1-week post implantation, mice were treated with NG25 (4 mg/kg/Day, intravenous injection), Trametinib (Tram, 1 mg/kg/Day, gavage), or combination for 15 days. n > 4 in each group. two-tailed t-test. **(E)** Image of tumors harvested in **D**. **(F)** Weight of tumors harvested in **D**. one-way ANOVA test. **(G)** NG25 synergizes with Trametinib to shrink CLU deficient Kras^G12D^ driven lung cancer in transgenic mouse model. Lsl-*Kras^G12D/+^* mice were treated with pSECC-sgCLU lentivirus by nasal inhalation. Tumor burdens were documented with CT. Mice were treated as indicated. NG25 (4 mg/kg/Day, intravenous injection), Trametinib (Tram, 1 mg/kg/Day, gavage), or combination for 12 days. n > 4 in each group. **(H)** relative tumor volume of mice of **G**.** (I)** Representative pathology images of mice of **G**.** (J)** Quantification of tumor numbers of mice of **G**. **(K)** Quantification of size of tumor in **G**. one-way ANOVA test on comparison of singlet treatment with the combo treatment. All the data plotted are mean ± s.e.m., ***P < 0.005, ****P < 0.0001, n = 3

Our results have shown that TAK1 inhibition may synergize with existing drug to treat CLU deficient lung cancer patients. Indeed, correlation between lower CLU expression and stronger sensitivity of NSCLC cells to TAK1 inhibition was confirmed when comparing NSCLC cell lines against their corresponding CLU-knockdown derivatives (Figure S6A-F). Earlier, we reported that MEK1/2 inhibitor partially regressed mutant Kras driven lung cancers 41. In our pilot CCK8 assay experiment, we found that while singlet drug treatment with TAK1 inhibitor (NG25) or MEK1/2 inhibitor (Trametinib) partially inhibited the growth rate of H358, combinational treatment almost completely blocked the cell growth (Figure 6A). Likewise, we also observed similar effect on colony formation in 2-D plates (Figure 6B -C).

We then went on to evaluate the treatment effect of combination of NG25 and Trametinib *in vivo*. To this end, we subcutaneously (s.c.) inoculated Hop62-shCLU cell in nude mice and randomized them for treatment with vehicle, NG25, Trametinib or combination of NG25 and Trametinib (designated combo) when tumors reached a volume of around 150 mm^3^. Interestingly, we found that while NG25 or Trametinib singlet treatment slowed down the growth rate of Hop62-shCLU xenograft tumors, combo treatment shrank the tumor (Figure 6D-E). Consistently, we observed that the weight of tumors in combo treated cohort was significantly lower than that of vehicle, NG25 or Trametinib treated cohort by the end of experiment (Figure 6F). Furthermore, combo treatment significantly inhibited proliferation and increased the apoptosis of the Hop62-shCLU tumors as revealed by Ki67 and Caspase3 immunochemistry (Figure S6G-I).

Mouse models of autochthonous lung cancer faithfully recapitulate the clinical course of lung cancer development and microenvironment of lung cancer patients. We then tested combination treatment on *CLU* knockout mouse model. For this purpose, we generated a cohort of K-CLU mice (see Figure 1 for methods and tumor pathology) and randomized them for treatment with NG25, Trametinib or combo after documenting tumor burden through CT imaging. As expected, we saw significant tumor growth in all groups of mice during the initial 20 days of vehicle treatment. Interestingly, CT imaging revealed dramatic tumor shrinkage in combo treated group in comparison to partial regression of tumor in NG25 or Trametinib treated group (Figure 6G-H). Consistently, pathological analysis revealed significantly lower tumor numbers and smaller tumor nodules in the cohort of combo treatment (Figure 6I-K). In line with this, we found occasional foci of tumor nodules with intra-tumoral spaces, thickened alveolar wall and slight fibrosis in NG25 or trametinib treated mice, all of which are much heavier in combo treated mice (Figure 6I), indicative of drastic remodeling process to replace previous tumor area. To test the translational significance of our combinational treatment in clinic, we assayed toxicity through blood biochemical assay to analyze the damage of liver (ALT and AST), kidney (CREA and UREA) and heart (LDH) of healthy C57J/B6 mice treated with the above dosing scheme. Our results revealed no significant toxicity in singlet- or combo- treated mice (Figure S6J-N). Taken together, our data showed unique sensitivity of *CLU* deficient lung cancer cells to TAK1 inhibitors.

## Discussion

Our current work highlighted an interesting concept: loss of function of a TSG rewired the signaling network and created an Achilles' heel in cancer cells, which could be exploited in cancer precision medicine. In case of *CLU*, its inactivation resulted in hyperactivation of TAK1- NF-κB signaling axis and TAK1 inhibitor exhibited exquisite cytotoxicity to *CLU* deficient lung cancer cells.

CLU protein is reported to undergo an intricate process of post-translational maturation. The N-terminal secretory signal peptide of 22 aa is cleaved off from CLU precursor, which is subsequently cleaved between residues Arg227-Ser228 to generate an α-chain and a β-chain. These 2 halves are assembled in antiparallel fashion to generate a heterodimeric molecule with five disulfide bridges 42. Consistent with previous report, we found that lung cancer cells harbored intracellular full-length and cleaved Clusterin and secreted cleaved protein 43.

Earlier report has shown that *CLU* expression promotes migration ability of NSCLC cells 44. However, clinical evidence for EMT/metastasis promoting activity of *CLU* is lacking in literature. Careful comparison between primary and metastatic NSCLC nodules are needed before clinical relevance of EMT/metastasis promoting activity of *CLU* can be solidified. Moreover, clinical evidence of significant association between higher *CLU* expression level and recurrence-free survival 45 suggests EMT/metastasis promoting activity of *CLU* detected *in vitro* to be an artifact. In contrast, clinical data, cell line data, and *in vivo* data in our current work strongly argue that *CLU* is a tumor suppressor in lung cancer. Likewise, significant correlation between higher *CLU* expression in clinic has been reported 45. In the same study, authors showed that higher *CLU* level significantly correlated with longer overall survival 45. Taken these evidences together, we conclude that *CLU* is a NSCLC tumor suppressor per se in lung cancer. Our work revealed that CLU negatively regulated TAK1 activity and the downstream NK-κB signaling. Of note, Bonacini et. al. have previously reported that NK-κB signaling pathway was negatively regulated by CLU in prostate cancer 46.

Considering facts that TGFBR1 directly recruits TRAF6^37^, that TAB2 mediates TAK1-TRAF6 interaction 38 and our observation that there exists a complex of TGFBR1/TRAF6/TAB2/TAK1 in lung cancer cells and 293T cells (Figures 3C-F & Figure S4A-E), our data argue the following working model for CLU's tumor suppressive role in NSCLC cells: CLU binds TGFBR1 and competes against TRAF6 for binding TGFBR1. CLU thus interferes recruitment of complex of TRAF6/TAB2/TAK1 by activated TGFBR1. We also envisioned that CLU binds TGFBR1 on a motif different from that bound by TRAF6.

Although clinical trials are been conducted targeting G12C mutant Kras (AMG 510 (NCT03600883) and MRTX849 (NCT03785249)) along with active research on siRNA delivery 47, KRAS mutation positive NSCLC remains the most difficult subtype of lung cancers to treat. Our current work suggested another strategy: concurrent dysfunction of TSGs in KRAS mutation positive tumors may create a new Achilles' heel. In case of *CLU* inactivation, lung cancer cells become uniquely sensitive to TAK1/NF-κB inhibition. Most strikingly, treatment data on cell line, xenograft tumor, and autochthonous lung cancer in transgenic mouse models showed that combinational inhibition of TAK1 and MEK1/2 effectively shrank lung cancer concurrently positive for KRAS mutation and *CLU* deficiency. Interestingly, combinational treatment with MEK/TAK1 inhibition was earlier found to be effective in eliciting apoptosis of Kras signaling dependent colon cancer cells through inhibition of mTOR, Wnt and NF-κB signaling 48.

TAK1 is an important mediator for TGFBRs' signaling. Of note, TGF-β signaling is involved in organizing an immunosuppressive micro-environment in tumor foci by inducing Tregs 49, 50. It is expected that TAK1 inhibition not only sensitizes NSCLC cells to targeting therapies, but normalizes immunity against tumors.

In short, here we identified *CLU* as a potent TSG in lung cancer. In activation of *CLU* leads to hyperactivation of TGFBR/TAK1 signaling axis. We show that this event is relevant to KRAS mutation positive NSCLC and that TAK1 inhibition synergizes with existing drugs to treat a portion of KRAS mutation positive NSCLC patients.

## Materials and methods

### Animal care and use

All mice were housed in a pathogen-free environment in Jinan University. Experimental protocols were approved by the Institutional Committee for Animal Care and Use at Jinan University and all animal work was performed in accordance with the approved protocol.

### Data base analysis of CLU expression in human lung cancer and correlation between expression of CLU and TAK1-NF-κB target genes

For comparison the CLU level between lung adenocarcinoma against para-tumoral tissues with GEO database. We downloaded the data from the NCBI GEO data sets (GSE10072 and GSE7670 with probe set 208792_s_at). In addition, we also compared CLU expression between normal tissue, para-tumoral and primary tumor tissues was performed with “**Compare tumor vs normal within or across tissue types**” function of UCSC Xena (http://xena.ucsc.edu/compare-tissue/) 51. The data base includes 1410 lung cancer samples from UCSC RNA-seq Compendium, where The Cancer Genome Atlas (TCGA) and the Genotype-Tissue Expression (GTEx, normal tissue of individuals without cancer) were re-aligned to hg38 genome by the same RNA-seq pipeline. The 'RSEM norm_count' dataset which was normalized by the upper quartile method was been chosen for the gene expression comparison. For correlation analysis between CLU and TAK1, IL1b, IL6 in lung adenocarcinoma patients, we acquired the gene expression data from TCGA database by searching UCSC Xena and the gene expression correlation was analyzed by GraphPad 6.0. For the expression specificity analysis of the CLU with Kras mutant in lung adenocarcinoma patients, patients were divided into *CLU* high and low expression groups according to the median of *CLU* expression. The expression specificity between the patients with Kras mutation with high or low *CLU* expression was analysis with Fisher' exact test, *P* < 0.05 is considered as significant correlation. Expression correlation analysis between CLU and IL1B or IL6 in these Kras mutant patient was analyzed by performed GraphPad 6.0. All the original data could be found in Table S1.

### Survival curve analysis

The survival curve analysis of *CLU* expression in lung cancer patients was using Kaplan-Meier-Plotter (http://kmplot.com/analysis/index.php?cancer=lung&p=service) by searching the TGCA database. Total and Stage I lung cancer patient were analyzed respectively in this study.

### Cell culture and cell engineering

Hop62 (KRAS G12C), NCI-H460 (KRAS G61H), A549 (KRAS G12S), H358 (KRAS G12C), HCC827 (EGFR G746-A750 deletion), H1975 (EGFR T790M and L858R), EKVX (non-KRAS), Hop92 (non-KRAS), H3255 (non-KRAS), H446 (non-KRAS) and H322 (non-KRAS) were purchased from ATCC (American Typical Culture Collection, Manassas, VA, USA). PC9 (EGFR G746-A750 deletion) and HEK293T were kindly provided by Dr. Kwok-Kin Wong (The Helen and Martin Kimmel Center for Stem Cell Biology, NYU). All the NSCLC cell lines were cultured in RPMI-1640 with 10% fetal bovine serum (FBS, Gibco, Life Technologies, Carlsbad, CA, USA). HEK293T cell was cultured in DMEM with 10% FBS. To generate the *CLU* knockdown NSCLC cell lines (shCLU), Hop62, H460, A549 and EKVX cell lines were infected with lentivirus packaged from pLKO-puro vector harboring shRNA sequence targeting *CLU* gene. To generate cell lines for Dox-inducible over-expression of *CLU*, H460, A549, EKVX and HEK293T cell were infected with lentivirus coding CLU (packaged from pLVX-tetone-CLU-puro vector). For CLU re-expression, Hop62 cell lines were infected with lentivirus encoding shRNA-resistant *CLU* gene (packaged from pLVX-tetone-zeo vector). To generate cell lines for Dox-inducible *TAK1* knockdown, Hop62-shCLU cells were infected with lentivirus packaged from pLKO-tet-zeo vector harboring shRNA targeting *TAK1* mRNA. Cells were selected with puromycin (2 μg/mL) for one week or zeomycin (300 μg/mL) for one week in appropriate cases.

### Bimolecular fluorescence complementation (BiFC) assay

293T-tet-CLU cells were co-transferred with 250 ng each of pCAG-TGFBR1-C-luc and pCAG-TRAF6-Nluc; or pCAG-TAB2-Nluc and pCAG-TAK1-Luc; pCAG-TAK1-CLUc and pCAG-TRAF6-Nluc; or pCAG-TAK1-CLUc and pCAG-TAB2-Nluc respectively in 24 well plate. 50 ng Renilla vector was also co-transferred in all the above experiments as the transfection efficiency control. 1 μg/mL of Dox was added to induce CLU expression in 293T-tet-CLU cell. Fluorescence detection was performed with the Dual Luminescent Kit and the Microplate Reader.

### Mouse treatment

All the transgenic mice were C57BL6 background about 6-8 weeks old with no restrictions on sex. To generate Kras^G12D^/CLU-/- (designated K-CLU) transgenic mice model, *CLU* sgRNAs were cloned into pSECC vector which co-expressing Cre and CRISPR/CAS9 (generously provided by F.J. Sanchez-Rivera and T. Jacks, Koch Institute for Integrative Cancer Research at MIT). Lentivirus of pSECC-sgCLU was packaged in 293T, validated by infecting NIH-3T3 cells and administered into lsl-Kras^G12D^ mice through nasal instillation. pSECC-TdTomato recombinant lentivirus was used as control (designated K-Ctl). Lung tumor formation in K-CLU mice were compared to K-Ctl mice 13 weeks post-infection. To generate lung cancer transgenic mice model for Dox induced *CLU* expression, lentivirus of pLVX-tet-CLU was packaged in 293T, validated by infecting NIH-3T3 cells and administered nasally into Tet-Kras^G12D^/CC10rtTA mice, Tet-EGFR L858R/CC10rtTA mice or Tet-EML4-ALK/CC10rtTA mice, respectively. pLVX-tet-mCherry virus was used as control. The lung tumor burdens were recorded through computed tomography scan (CT, PINGSENG Healthcare) after 2 around mouths of Dox diet feeding. Mice were treated with NG25 (intraperitoneal injection, i.p, 4 mg/kg/Day), Trametinib (gavaged, 1 mg/kg/Day), NG25 and Trametinib combination or vehicle. The tumor size of the mice was analysis with ImageJ. Tumor burden comparison analysis followed our previous published study 29.

For xenograft tumor model, 2 × 10^6^ engineered cancer cells were subcutaneously implanted into the two flanks of the nude mice. Tumors were left to fix for 1 week. Tumor-bearing mice were treated with NG25 (intraperitoneal injection, i.p, 4 mg/kg/Day), Trametinib (gavaged, 1mg/kg/Day), NG25 and Trametinib combination or vehicle. 15 days after treatment, the mice were sacrificed and the tumors burden was dissected. The tumor volume is calculated by the formula: volume = (width)^2^ × length × 0.5.

### Statistical analysis

All the data were presented as mean values ± s.e.m. Differences between two groups compared with unpaired two-tailed t-test, while multiple comparisons was used one-way ANOVA with Bonferroni post hoc test. Statistical analyses were performed with GraphPad Prism 6.0, *P* < 0.05 was deemed to be statistically significant.

## Supplementary Material

Supplementary figures.

Supplementary table S1.

Supplementary table S2.
