# Supplementary material for: Inactivation of tumor suppressor gene Clusterin leads to hyperactivation of TAK1-NF-κB signaling axis in lung cancer cells and denotes a therapeutic opportunity
Source: Theranostics. 2020 Sep 16;10(25):11520–34. doi: 10.7150/thno.44829 (PMC7545994; doi:10.7150/thno.44829)
Supplement: Supplementary file 1 — Supplementary figures. [file thnov10p11520s1.pdf]

Inactivation of tumor suppressor gene Clusterin leads to hyperactivation of TAK1-NF-κB signaling axis in lung cancer cells and denotes a therapeutic opportunity

Zhipeng Chen[1*], Zhenzhen Fan[1*], Xiaowei Dou[2*], Qian Zhou[1*], Guandi Zeng[1*], Lu Liu[1], Wensheng Chen[1], Ruirui Lan[1], Wanting Liu[1], Guoqing Ru[3#], Lei Yu[4#], Qing-Yu He[1#] & Liang Chen[1#]

Running title: TAK1 inhibitor synergizes to treat CLU deficient NSCLC

Material and methods

Plasmids

Human *CLU* cDNA (TRCN0000491595, MISSION® TRC3 Human ORF Clone Collection) was subcloned into lentivirus vector pLVX-tetone3GS-puro (purchased from Clontech Laboratories, Inc.) or pLVX-tetone3GS-mcherry (changed the puromycin resistant gene to mcherry of pLVX-tetone3GS-puro vector in house), while CLU-resisted shCLU gene was subcloned into pLVX-tetone3GS-zeomycin (changed the puromycin resistant gene to zeomycin of pLVX-tetone3GS-puro vector in house). For co-IP experiment, human TAK1 (with Flag tag), TGFBR1 (with Myc tag) and CLU (with 3×Flag tag) were subcloned into pcDNA3.1 vector (purchased from Life Technologies). For Bimolecular fluorescence complementation (BiFC) assay, TAK1-N-luc or TAK1-C-luc, TGFBR1-C-luc, TRAF6, and TAB1- N-luc or TAB2-N-luc were subcloned into pCAGiN (Plasmid#13461, Addgene,) vector. For gene knockdown experiments, shRNA fragments were cloned into pLKO.1-puro (Plasmid#8453, Addgene) and Tet-pLKO-puro (Plasmid#21915, Addgene) vector. shRNAs were purchased from the Sigma Mission shRNA Library: shCLU (TRCN0000078611), shTAK1 (TRCN0000001554).

To generate TGFBR1 truncations, pcDNA3.1-TGFBR1-myc plasmid was used as template and three truncations of TGFBR1 (DEL2: deleted 218-318aa; DEL3: deleted 318-418aa; DEL4: deleted 124-217aa) were cloned by QuikChange II Site-Directed Mutagenesis Kit (Catalog #200523, Agilent) following manufacturer's instruction with following primers:

DEL2 Forward:

5’-TTGATTACTTAAACAGATACTTCAAACGTGCTGACATCTATGC-3’

DEL2 Reverse: 5’- GTATCTGTTTAAGTAATCAAAAAGGGATCCATG-3’

DEL3 Forward: 5’-

ATATGAAACATTTTGAATCCAGTCAACAGGAAGGCATCAAAATG-3’

DEL3 Reverse: 5’- GGATTCAAAATGTTTCATATTTATGGAATCATCG-3’

DEL4 Forward: 5’-

CATTGCTTGTTCAGAGAACATACACAGTTACTGTGGAAGGAATG-3’

DEL4 Reverse: 5’-

TGTTCTCTGAACAAGCAATGGTAAACCAGTAGTTGGAAGTTCTAT-3’

Cell proliferation assay

Cells activity was monitored with CCK8 reagent every day following manufacturer’s instruction (Lot. PJ762, DOJINDO Laboratories). 800 cells were seeded in 96 wells plate and culture overnight. For Dox inducible cells, Dox was added in medium to the final concentration of 1 ug/mL.

Plate and soft-agar colony formation assay

Colony formation assay was performed following our procedures[1]. Briefly, 200 cells/well were seeded in 6 wells plate and cultured for indicated days. Cell colonies were fixed and stained with 0.5% crystal violet in methyl alcohol. Images of cell clones were acquired and colonies were counted with ImageJ and the statistical analyzes was using GraphPad Prism 6.0.

For soft-agar colony formation assay, the agar gel was loaded in 6 well plates with the concentration of the gel was 0.6% in lower and 0.35% in upper. 200 cells/well were mixed with the 0.35% upper gel before loaded into the well. Colonies images were acquired with inverted microscope and the colonies with the diameter over 200 μm were counted. Statistical analyzes was using GraphPad Prism 6.0.

Protein extraction, immunoblotting and immunoprecipitation

We conducted protein extraction followed procedures we published earlier[1]. Briefly, whole cell lysates were extracted by RIPA lysis buffer (P0013B, Bayotime, China) along with protease and phosphatase inhibitor cocktail (4693132001& 4906837001, Roche, Basel, Switzerland). Protein concentration was determined with BCA assay (Prrsis). Proteins (50–100 μg) were subjected to SDS–polyacrylamide gel electrophoresis. Separated proteins were electrophoretically transferred onto polyvinylidene difluoride (PVDF) membranes (Millipore, Billerica, MA, USA) and immunoblotted with anti-CLU (42143S, 1:1000, Cell Signaling Technology, CST), -pTAK1 (4508S, 1:500, CST), TAK1 (5206S, 1:1000, CST), Fibronectin (sc-271098, 1:1000, Santa Cruz Biotechnology),-Flag (F1804, 1:2000, Sigma), -myc (M4439, 1:2000, Sigma), TGFBR1 (ab31013, 1:500, Abcam), -pP65 (ab86299, 1:1000, Abcam), -P65 (ab16502, 1:1000, Abcam), -pIKB (ab133462, 1:1000, Abcam), -IKB(ab32518, 1:1000, Abcam), -p105/p50 (ab32360, 1:1000, Abcam), -GAPDH (ab128915, 1:2000, Abcam), -LaminB1 (ab16048, 1:1000, Abcam), or β-actin (A5316, 1:2000, Sigma). Immunoreactive proteins were visualized using ECL Western

Blotting Substrate (Millipore).

Immunoprecipitation experiment was performed as previous published[2]. In brief, cells transferred as indicated plasmids were lysed with lysis buffer (9803, CST). Cell lysate was incubated with antibody at 4 ℃ overnight, then added the agar beads purchased from Santa cruz for another 4 h, wash the beads 5 times before eluted by SDS protein loading buffer at 95 ℃. The protein was further immunoblotted.

RNA extraction and reverse-transcription PCR

Total RNA was extracted using Trizol reagent (15596-018, Invitrogen) following the instruction manual. For reverse-transcription, 2 μg total RNA was reverse-transcribed to cDNA with OneScript® Plus cDNA Synthesis Kit (abm, Canada). Human ACTB (β-actin) gene was used as an internal control. Real-time PCR was performed with BioRad CFX Connect Real-time PCR machine (Bio-rad) and Universal SYBR green reagent (BioRed) with the following primers:

CLU-Forward: 5’-TGCGGATGAAGGACCAGTGTGA-3’,

CLU-Reverse: 5’-TTTCCTGGTCAACCTCTCAGCG-3’,

FN1-Forward: 5’-ACAACACCGAGGTGACTGAGAC-3’,

FN1-Reverse: 5’-GGACACAACGATGCTTCCTGAG-3’,

IL1B-Forward: 5’-ATGATGGCTTATTACAGTGGCAA-3’

IL1B-Reverse: 5’- GTCGGAGATTCGTAGCTGGA-3’

TNF-Forward: 5’- CCTGTAGCCCACGTCGTAGC-3’

TNF-Reverse: 5’- AGCAATGACTCCAAAGTAGACC-3’

JUN-Forward: 5’- CCTTGAAAGCTCAGAACTCGGAG-3’

JUN-Reverse: 5’- TGCTGCGTTAGCATGAGTTGGC-3’

ACTB-Forward: 5’- ACGTGGACATCCGCAAAG-3’

ACTB-Reverse: 5’- GACTCGTCATACTCCTGCTTG-3’

TAK1-Forward: 5’-CAGAGCAACTCTGCCACCAGTA-3’

TAK1-Reverse: 5’- CATTTGTGGCAGGAACTTGCTCC-3’

COL1A1-Forward: 5’- GATTCCCTGGACCTAAAGGTGC-3’

COL1A1-Reverse: 5’- AGCCTCTCCATCTTTGCCAGCA-3’

COL4A1-Forward: 5’- TGTTGACGGCTTACCTGGAGAC-3’

COL4A1-Reverse: 5’- GGTAGACCAACTCCAGGCTCTC-3’

Splicing variants analysis of CLU

CLU alternative splice variants were determined according to paper by Leskov et.al. [3]. RNA samples extracted from patients’ lung cancer and lung cancer cells line was reverse-transcribed and PCR was amplified with following primers: hCLU-F : 5’-ACAGGGTGCCGCTGACC-3’; hCLU-R : 5’-GAGCTCCTTCAGCTTTGTCTCTG-3’. PCR products were separated by high-resolution agarose-gel electrophoresis analysis. sCLU product is 340 bp while nCLU is 220 bp. The PCR product was further confirmed by sequencing.

Immunohistochemistry

IHC staining was performed as previously described[4]. The following antibodies were used: CLU (sc-166831, Santa cruz), Ki67 (NCL-Ki67p; Leica Biosystems), and

Cleaved Caspase-3 (9661; CST). The staining of the antibodies was analyzed with ImageJ and the quantification was performed by GraphPad Prism 6.0.

Blood biochemical assay

Blood biochemical assay was performed by Wuhan servicebio technology CO., LTD with fully automatic biochemical analyzer (Chemray 800, Rayto, Shenzhen, China). Serum of the mice with indicated treatment were acquired. Alanine aminotransferase (ALT), Aspartate amino transferase (AST), Urea nitrogen (UREA), Creatinine (CREA) and Lactic dehydrogenase (LDH) were analyzed.

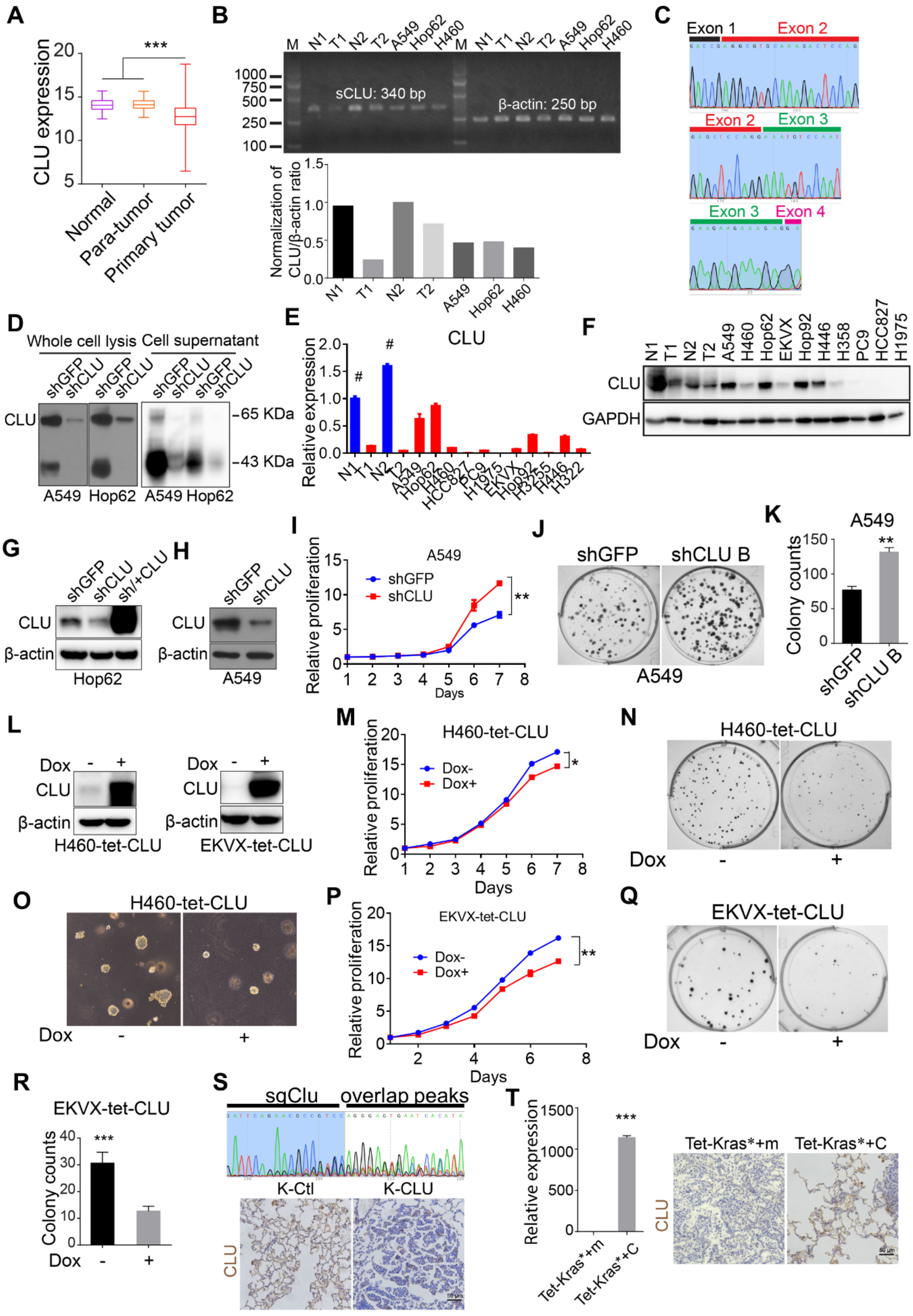
A
CLU expression
***
Normal
Para-tumor
Primary tumor
B
M N1 T1 N2 T2 A549 Hop62 H460 M N1 T1 N2 T2 A549 Hop62 H460
1000
750
500
250
100
sCLU: 340 bp
β-actin: 250 bp
Normalization of CLU/β-actin ratio
C
Exon 1
Exon 2
Exon 2
Exon 3
Exon 3
Exon 4
D
Whole cell lysis
Cell supernatant
shGFP shCLU shGFP shCLU shGFP shCLU shGFP shCLU
CLU
-65 KDa
-43 KDa
A549 Hop62 A549 Hop62
E
CLU
Relative expression
N1 T1 N2 T2 A549 Hop62 H460 HCC827 PC9 H1975 EKVX Hop92 H3255 H446 H322
F
N1 T1 N2 T2 A549 H460 Hop62 EKVX Hop92 H446 H358 PC9 HCC827 H1975
CLU
GAPDH
G
shGFP shCLU sh/+CLU
CLU
β-actin
Hop62
H
shGFP shCLU
CLU
β-actin
A549
I
A549
shGFP
shCLU
Relative proliferation
Days
**
J
shGFP
shCLU B
A549
K
A549
Colony counts
**
shGFP
shCLU B
L
Dox - +
CLU
β-actin
H460-tet-CLU
EKVX-tet-CLU
M
H460-tet-CLU
Dox-
Dox+
Relative proliferation
Days
*
N
H460-tet-CLU
Dox - +
O
H460-tet-CLU
Dox - +
P
EKVX-tet-CLU
Dox-
Dox+
Relative proliferation
Days
**
Q
EKVX-tet-CLU
Dox - +
R
EKVX-tet-CLU
Colony counts
***
Dox - +
S
sgClu
overlap peaks
K-Ctl
K-CLU
CLU
T
Relative expression
***
Tet-Kras*+m
Tet-Kras*+C
CLU

Figure S1. *CLU* is an essential tumor suppressor in lung tumorigenesis. (**A**) Comparison of *CLU* expression between normal, para-tumor and primary tumor. Box plots showing the median expression level of normal (n = 288), para-tumoral (n = 109), and primary tumor (n = 1011) respectively. Statistic with one-way ANOVA test. (**B**) Semi-quantitative high-resolution agarose-gel electrophoresis of PCR products determining CLU splicing variants (upper) and relative quantification of CLU (below). RNA samples of para-tumoral (N), tumor (T) from patients and cell lines were reverse transcribed into cDNA. PCRs were performed with a single pair of primes capable of amplifying all variants of CLU (methods) which can distinguish by size reveal in agarose gel electrophoresis: 340 bp for sCLU; 220 bp for cCLU (not detected). (**C**) Sanger sequencing confirmed authenticity of sCLU. (**D**) western analysis of expression CLU and its proteolytic products in whole cell lysate or concentrated cell culture supernatant. Lung cancer cell lines were culture for 48 h and cell supernatant was collected with ultrafiltration device. Whole cell lysate and concentrated supernatant protein were separated with SDS-PAGE followed by immunoblotting with CLU antibody. (**E**) Evaluation of CLU expression with qPCR among para-tumor and primary tumor tissue of lung cancer patient and multiple lung cancer cell lines; N: para-tumor, T: primary tumor. CLU expression of para-tumoral tissues was compared with primary tumor or lung cancer cell lines, respectively. $P < 0.0001$, n = 3. (**F**) Protein levels of CLU in lung cancer para-tumoral, primary tumoral tissues and lung cancer cell lines indicated. (**G**) Evaluation of CLU expression in CLU knockdown and CLU re-expression in engineered Hop62 cells. (**H**) CLU knockdown efficiency in

A549 cell. (**I**) Cell proliferation assay of A549 in indicated groups. (**J**) and (**K**) 2-D plate colony formation capacity assay of A549 **J** and quantification **K**. (**L**) CLU expression was induced with Dox in H460 and EKVX cell. (**M**), (**N**) and (**O**) Cell proliferation, 2-D plate colonies formation and soft agar colonies formation assay of H460-tet-CLU cells. (**P**), (**Q**) and (**R**) Cell proliferation, 2-D plate colonies formation and colonies quantification of EKVX-tet-CLU cells. (**S**) Sanger sequencing confirmed the knockout efficiency of CLU by lentiviruses and IHC staining of CLU in lung section of K-Ctl mice and K-CLU mice. (**T**) expression of human CLU in lungs of Tet-Kras* + m or Tet-Kras* + C transgenic mice revealed through RT-PCR (left) and IHC staining (right). All the data are shown as mean ± SEM, statistics with two-tailed t-test. ***P*<0.005, ****P* < 0.0001, n = 3.

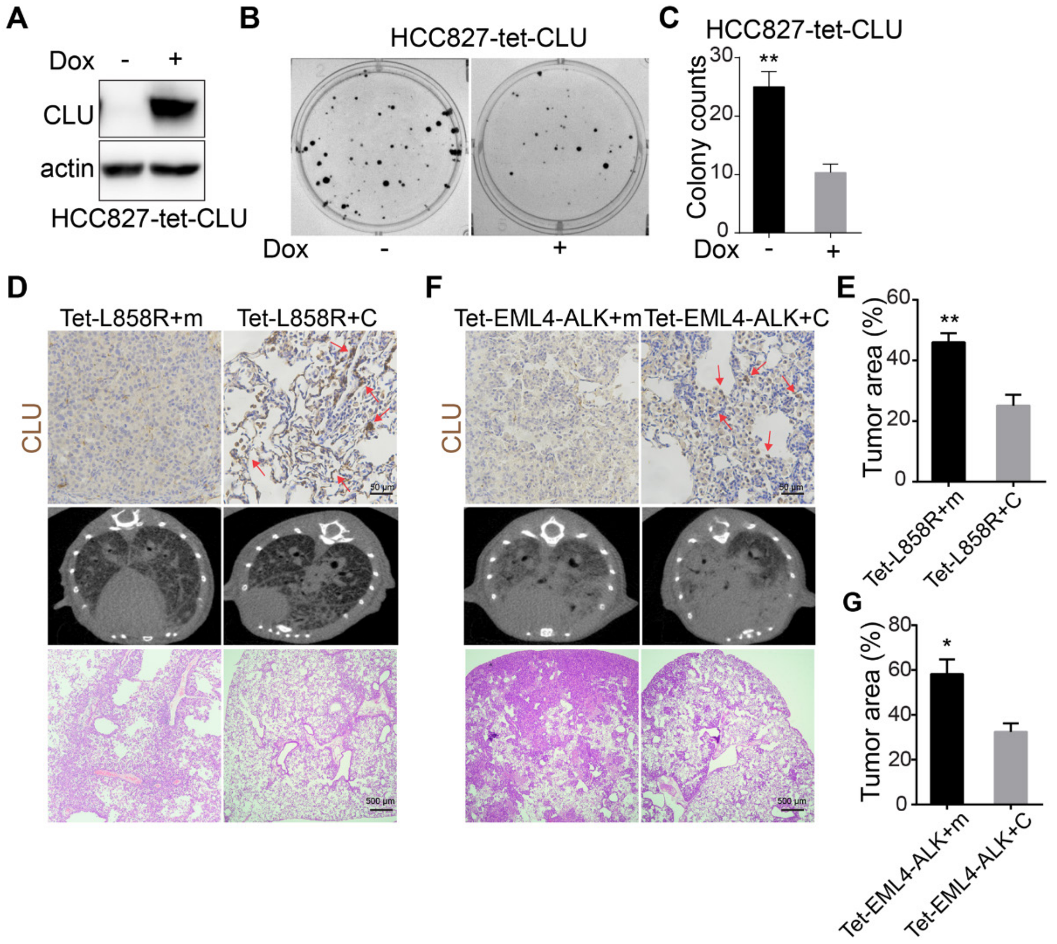

A
Dox - +
CLU
actin
HCC827-tet-CLU
B
HCC827-tet-CLU
Dox - +
C
HCC827-tet-CLU
Colony counts
30
20
10
0
**
Dox - +
D
Tet-L858R+m
Tet-L858R+C
CLU
50 μm
500 μm
F
Tet-EML4-ALK+m
Tet-EML4-ALK+C
CLU
50 μm
500 μm
E
Tumor area (%)
60
40
20
0
**
Tet-L858R+m
Tet-L858R+C
G
Tumor area (%)
80
60
40
20
0
*
Tet-EML4-ALK+m
Tet-EML4-ALK+C

Figure S2. CLU plays tumor suppressive role in lung cancers driven by mutant EGFR and EML4-ALK. (**A**) Western blot evaluation of the Dox inducible expression of CLU in HCC827-tet-CLU cell line. (**B**) 2-D plate colony formation assay of HCC827-tet-CLU. (**C**) Bar graph of the result shown in **B**. (**D**) Impact of CLU expression on tumor formation in CC10rtTA /Tet-EGFR L858R bitransgenic mice. CC10rtTA /Tet-EGFR L858R bitransgenic mice were infected with lentivirus harboring Tet-mCherry (Tet-L858R + m, serving as negative control) or Tet-CLU (Tet-L858R + C) were fed with Dox diet for 2 months. Upper panel: IHC staining of CLU in lung of the transgenic mice; Middle panel: Computed tomography; Lower panel: hematoxylin and eosin (H&E) staining of lungs of transgenic mice. (**E**) Bar graph of percentage of tumor area relative to the lung section of the transgene mice. **D**. (**F**) Impact of CLU expression on tumor formation in CC10rtTA /Tet-EML4-ALK bitransgenic mice. Mice infected with lentivirus harboring Tet-mCherry (Tet-EML4-ALK+m, serving as negative control) or Tet-CLU (Tet-EML4-ALK + C) were fed with Dox diet for 2 months. Upper panel: IHC staining of CLU in lungs of the transgenic mice; Middle panel: Computed tomography images of transgenic mice; Lower panel: H&E staining of lung sections. (**G**) Bar graph of percentage of tumor area relative to the lung section of the transgene mice in F. *$P < 0.05$ **$P < 0.005$; Data plotted are mean ± s.e.m.; n = 3.

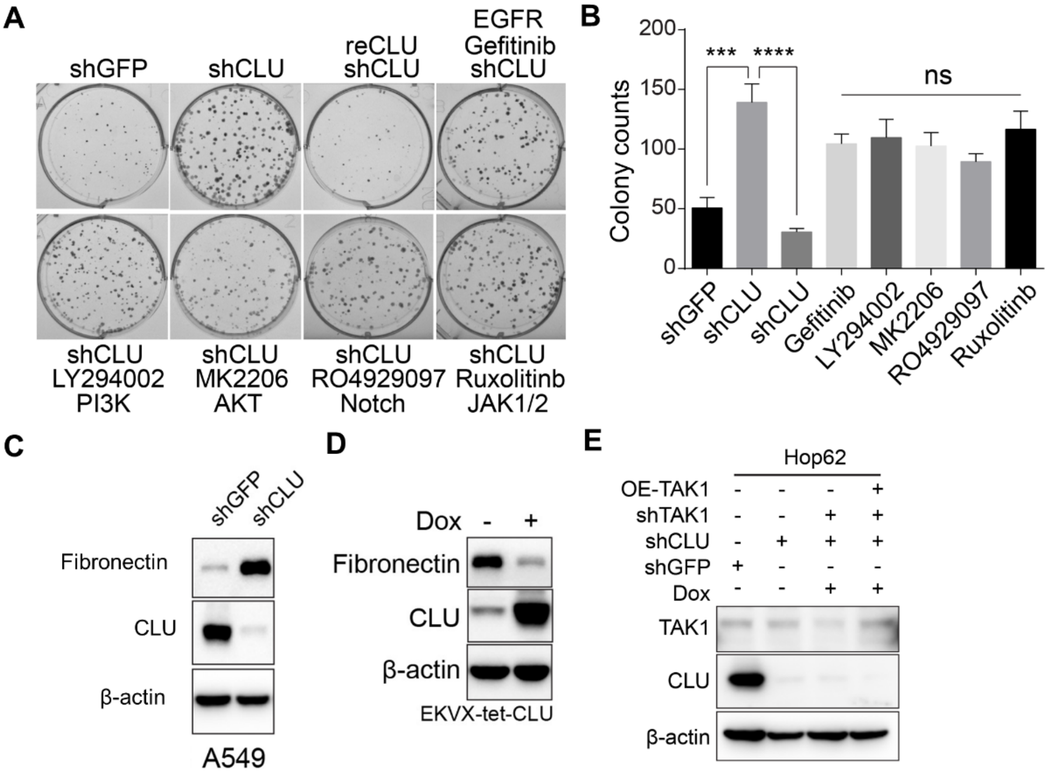
A
shGFP
shCLU
reCLU
shCLU
EGFR
Gefitinib
shCLU
shCLU
LY294002
PI3K
shCLU
MK2206
AKT
shCLU
RO4929097
Notch
shCLU
Ruxolitinb
JAK1/2
B
Colony counts
200
150
100
50
0
***
****
ns
shGFP
shCLU
shCLU
Gefitinib
LY294002
MK2206
RO4929097
Ruxolitinb
C
shGFP
shCLU
Fibronectin
CLU
β-actin
A549
D
Dox
-
+
Fibronectin
CLU
β-actin
EKVX-tet-CLU
E
Hop62
OE-TAK1
shTAK1
shCLU
shGFP
Dox
TAK1
CLU
β-actin

Figure S3. CLU knockdown enhances TAK1 signaling. (**A**) TAK1 inhibitor specifically inhibited colony formation of CLU knockdown lung cancer cells. Hop62-shCLU cells were treated with indicated inhibitors Gefitinib (2.5 μM), LY294002 (2.5 μM), MK2206 (2.5 μM), RO4929097 (2.5 μM), Ruxolitinib (2.5 μM). (**B**) Quantification of colony numbers with indicated treatments. Significance shown are calculated on indicated groups against Hop62-shCLU groups. Data plotted are mean ± s.e.m.****P*<0.0001, ns, not significant. one-way ANOVA test. (**C**) and (**D**) Western blot evaluated the Fibronectin and CLU level in indicated cells. (**E**) Western blot showing TAK1 knockdown in Hop62-shCLU cells and re-expression in Hop62-shCLU/tet-shTAK1 cells.

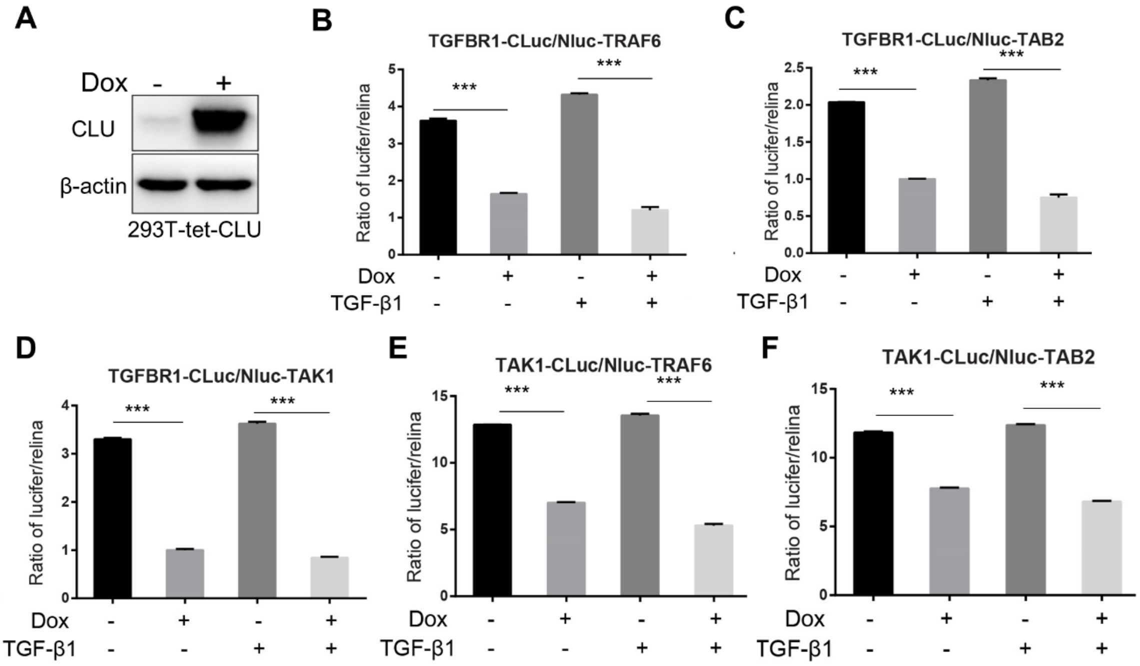

A
Dox - +
CLU
β-actin
293T-tet-CLU
B
TGFBR1-CLuc/Nluc-TRAF6
Ratio of lucifer/relina
***
***
Dox - + - +
TGF-β1 - - + +
C
TGFBR1-CLuc/Nluc-TAB2
Ratio of lucifer/relina
***
***
Dox - + - +
TGF-β1 - - + +
D
TGFBR1-CLuc/Nluc-TAK1
Ratio of lucifer/relina
***
***
Dox - + - +
TGF-β1 - - + +
E
TAK1-CLuc/Nluc-TRAF6
Ratio of lucifer/relina
***
***
Dox - + - +
TGF-β1 - - + +
F
TAK1-CLuc/Nluc-TAB2
Ratio of lucifer/relina
***
***
Dox - + - +
TGF-β1 - - + +

Figure S4. CLU competes against TAK1 for binding TGFBR1. (**A**) Western blot evaluation of Dox inducible CLU expression in 293T cells. (**B**) and (**C**) BiFC assay evaluating the impact of CLU on interaction between TGFBR1-C-luc and N-luc-TRAF6 or N-luc-TAB2. (**D**) BiFC assay evaluated impact of CLU on interaction between TGFBR1-C-luc and N-luc-TAK1. (**E**) and (F) BiFC assay evaluated the impact of CLU on interaction between TAK1-C-luc and N-luc-TRAF6, or TAB2. 293T-Tet-CLU cells were co-transfected with indicated expressing plasmids. Renilla was co-transfected as reference. Data are shown as mean ± SEM, statistics with two-tailed t-test. ***$P < 0.0001$, n = 3.

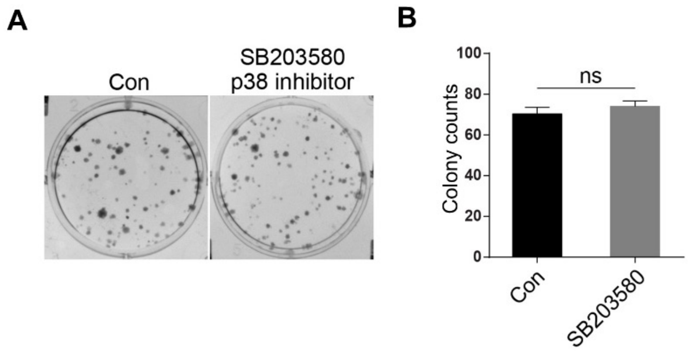
A
Con
SB203580
p38 inhibitor
B
Colony counts
100
80
60
40
20
0
ns
Con
SB203580

Figure S5. TAK1-NF-κB pathway mediates growth-promoting effects in CLU-deficient lung cancer cells. (**A**) 2-D plate colony formation of Hop62-shCLU cells treated with or without p38 inhibitor SB203580. (**B**) Quantification of cell colonies. data plotted are mean ± s.e.m. ns, no significant, two tail student t-test.

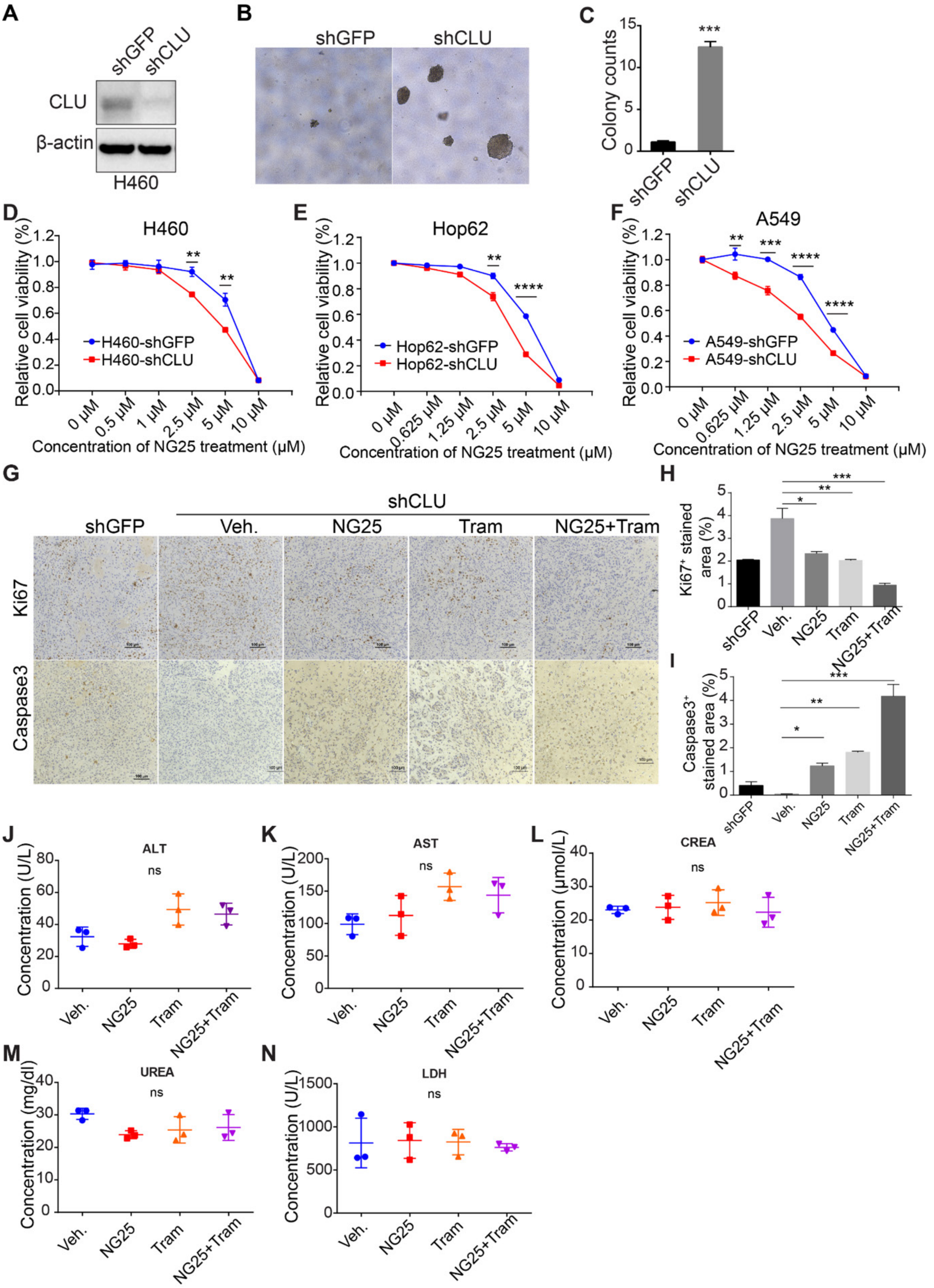
A
shGFP
shCLU
CLU
β-actin
H460
B
shGFP
shCLU
C
Colony counts
***
shGFP
shCLU
D
H460
Relative cell viability (%)
H460-shGFP
H460-shCLU
Concentration of NG25 treatment (μM)
E
Hop62
Hop62-shGFP
Hop62-shCLU
F
A549
A549-shGFP
A549-shCLU
G
shCLU
shGFP
Veh.
NG25
Tram
NG25+Tram
Ki67
Caspase3
H
Ki67⁺ stained area (%)
I
Caspase3⁺ stained area (%)
J
ALT
ns
Concentration (U/L)
K
AST
L
CREA
Concentration (μmol/L)
M
UREA
Concentration (mg/dl)
N
LDH

Figure S6. TAK1 inhibitor synergizes with existing therapeutics to treat CLU deficient lung cancer. (**A**) knockdown efficiency of CLU in H460-shCLU cell. (**B**). CLU knockdown increased capacity of H460 cells to form colonies in soft-agar culture. (**C**). Bar graph of result shown in **B**. (**D-F**). CLU knockdown enhanced sensitivity of lung cancer cells positive of Kras mutation to treatment of TAK1 inhibitors. CCK8 assay of control knockdown (shGFP) or CLU knockdown (shCLU) cells were conducted. (**G**) Representative immunochemistry image of Ki67 and Caspase3 in Hop62-shCLU tumor with indicated treatment. (**H**) and (**I**) Quantification of Ki67 and Caspase3 shown in **G**. All the data are shown as mean ± SEM, statistics with one-way ANOVA test, $^{*}P < 0.05$, $^{**}P < 0.005$, $^{***}P < 0.0001$, n = 3. (**J-N**). Toxicity of liver, kidney and heart were assayed with Alanine aminotransferase (ALT) and Aspartate amino transferase (AST); Creatinine (CREA) and Urea nitrogen (UREA); Lactic dehydrogenase (LDH). Healthy C57BL/6 mice were randomized into groups for treatment with Veh., NG25, Tram and combo. NG25 (4 mg/kg/Day, intravenous injection), Trametinib (Tram, 1 mg/kg/Day, intra gavage), or combination for 5 days. Blood was withdrawn from these mice, and serum were used for biochemical assay. ns, not significant, n = 3.

Reference

1. Gao L, Hu Y, Tian Y, Fan Z, Wang K, Li H, et al. Lung cancer deficient in the tumor suppressor GATA4 is sensitive to TGFBR1 inhibition. Nat Commun. 2019; 10: 1665.
2. Lu F, Zhou Q, Liu L, Zeng G, Ci W, Liu W, et al. A tumor suppressor enhancing module orchestrated by GATA4 denotes a therapeutic opportunity for GATA4 deficient HCC patients. Theranostics. 2020; 10: 484-97.
3. Leskov KS, Klokov DY, Li J, Kinsella TJ, Boothman DA. Synthesis and functional analyses of nuclear clusterin, a cell death protein. J Biol Chem. 2003; 278: 11590-600.
4. Wu Q, Tian Y, Zhang J, Tong X, Huang H, Li S, et al. In vivo CRISPR screening unveils histone demethylase UTX as an important epigenetic regulator in lung tumorigenesis. Proc Natl Acad Sci U S A. 2018; 115: E3978-E86.
